# Use of Technology to Access Health Information/Services and Subsequent Association With WASH (Water Access, Sanitation, and Hygiene) Knowledge and Behaviors Among Women With Children Under 2 Years of Age in Indonesia: Cross-sectional Study

**DOI:** 10.2196/19349

**Published:** 2021-01-14

**Authors:** Heidi Jane Niedfeldt, Emmalene Beckstead, Emily Chahalis, Mindy Jensen, Britton Reher, Scott Torres, Cut Novianti Rachmi, Hafizah Jusril, Cougar Hall, Joshua H West, Benjamin T Crookston

**Affiliations:** 1 Department of Public Health Brigham Young University Provo, UT United States; 2 RTI International Washington, DC United States; 3 Reconstra Jakarta Indonesia

**Keywords:** technology, defecation, handwashing, WASH, stunting

## Abstract

**Background:**

Water access, sanitation, and hygiene (WASH) remain a public health concern in Indonesia. Proper WASH practices can decrease risk of stunting, wasting, and disease in children under the age of 2.

**Objective:**

The purpose of our study is to examine if using technology to access health information and services among Indonesian women affects knowledge and behaviors regarding handwashing and defecation practices.

**Methods:**

Our study is an interview-based cross-sectional survey. Participants included 1734 mothers of children under 2 years of age. These women were randomly selected and interviewed as part of a 3-stage cluster sampling technique. Our study uses data regarding WASH knowledge which includes benefits of handwashing with soap, 5 critical times of handwashing, risks of open defecation, media of disease transmission, defecation locations, and risks of open defecation. Data regarding WASH behaviors were also included: handwashing with soap, type of latrine used at home, and where defecation took place. This investigation used adjusted and unadjusted logistic and linear regression models to determine differences in WASH outcomes between those who use technology to access health information and services and those who did not.

**Results:**

One result is that Indonesian women with children under 2 years of age who use technology to access health information and services are more likely to know the advantages of proper handwashing (odds ratio [OR] 2.603, 95% CI 1.666-4.067) and know the 5 critical times of handwashing (OR 1.217, 95% CI 0.969-1.528). Women who use technology to access health information are also more likely to know the risks of open defecation (OR 1.627, 95% CI 1.170-2.264) and use a type of toilet (such as a gooseneck or squat toilet) that limits risk (OR 3.858, 95% CI 2.628-5.665) compared to women who did not use technology to access health information.

**Conclusions:**

Using technology to access health information and services was associated with an increase in handwashing and defecation knowledge. In the future, promoting mothers of children under 2 years of age to access health information through technology might be used to increase handwashing and defecation knowledge as well as safe defecation practices. However, further research should be done to determine how technology may increase the frequency of recommended handwashing behaviors.

## Introduction

Water access, sanitation, and hygiene (WASH) remain a global public health concern. As of 2014, about 2.5 billion individuals worldwide did not use an improved sanitation facility (a sanitation facility that keeps human excreta and human contact hygienically separate) and about 1 billion of these individuals practice open defecation [1]. Additionally, about 700 million individuals did not have access to improved water (water that is protected from outside contamination such as fecal matter) [1]. In Indonesia about 30 million individuals practice open defecation [2].

WASH practices play an important role in the healthy development of children, especially those under the age of 2. In urban areas of East Jakarta, Indonesia, children living in a house with more sewage have a higher prevalence of diarrhea than those who live in houses with less sewage [3]. Diarrheal disease is the second leading cause of death among children under the age of 5 worldwide [4]. Households in 112 rural districts in India that had access to a toilet facility, compared with open defecation, had 39% reduced odds of childhood stunting in the first 24 months [5]. Stunting is of particular concern to child development as stunted children have reduced cognitive function, adult economic productivity, as well as increased mortality and morbidity [6,7]. Stunting is also a major challenge in Indonesia where approximately 37% of all children are stunted [8].

Individuals can obtain health information from a variety of traditional print, radio, or television media, which have been effective components of health communication interventions [9,10]. In recent years, interventions addressing health behaviors have used emerging technologies, starting with SMS text messages. For example, in a review of 13 studies addressing health disparities, researchers found that using SMS text messaging interventions can have positive short-term behavioral outcomes [11]. Cormick et al [12] found that 96% of women in urban Argentina would like to receive health information via SMS text messages about prenatal care. In addition to SMS text messages, the emergence of smartphones has increased opportunities for individuals to access health information more readily, which may influence dietary intake, reduce stunting rates, and address other health disparities in children. For instance, one study in the Changning District of Shanghai, China, found that 26.2% of pregnant mothers used an app to learn about nutrition and to record their diet [13]. A review of 4 studies of women in urban and rural low and lower middle-income countries (Indonesia, Kenya, and 2 in India) shows that mobile health interventions improve nutrient intake of pregnant women [10,14]. Specific to Indonesia, smartphone users are estimated to be more than 150 million, or approximately 56% of the population [15]. While smartphone ownership is highest in urban areas (71%), ownership in rural areas has grown rapidly over the past decade and was estimated at 42% in 2018. Chatting and SMS text messaging are the most commonly reported smartphone activities among Indonesians, followed by social media use, image and video searches, and gaming [16]. Household computer and laptop use for Internet access are comparatively low at just 31% in urban areas and 24% in rural areas [16].

While there is research about mobile phones, there is a lack of studies on using smartphones, tablets, and computers to access information about WASH and safe defecation practices to improve childhood health in rural Indonesia. The purpose of our study was to explore the relationship of accessing WASH-related knowledge using a smartphone, tablet, or computer with improvements in WASH knowledge and behaviors among women in Indonesia. In particular, our study sought to understand how accessing knowledge via a smartphone, tablet, or computer may impact safe defecation knowledge and practices.

## Methods

### Design

Our study included an analysis of cross-sectional data collected in rural Indonesia following the 2014-2018 National Nutrition Communication Campaign (NNCC) intervention and represents a collaborative effort between IMA (Interchurch Medical Assistance)–World Health, the University of Indonesia’s Center for Nutrition and Health Studies, and the Ministry of Health in Indonesia. The NNCC was designed to address the health condition of stunting that impacts numerous children in Indonesia. Of the 34 provinces in Indonesia, the NNCC utilized mass media campaigns, advocacy interventions, and interpersonal communication in 3 provinces, working in over 688 (approximately 74,000 nationwide) villages and 11 districts (approximately 7000 nationwide).

### Sample

The study sample consisted of 1734 mothers of children under 2 years of age from 3 rural districts (Banyuasin, Kubu Raya, and Katingan) located in 3 provinces (South Sumatra, West Kalimantan, and Central Kalimantan) in Indonesia. One district was randomly selected from each of the 3 provinces. A multilevel sampling strategy was used to construct the study sample. Within each of the 3 rural districts, 30 villages were randomly selected, and each represented a cluster unit. At a more local level, 4 subvillages were randomly selected from within each of the 30 villages, in each of the 3 districts. Finally, in each of these subvillages a list was compiled from a local health center of mothers of children under the age of 2 and 5 mothers were selected randomly from the list from each subvillage. After using the formula for a hypothesis testing between 2 population proportions, the target sample size from each of the 3 districts was determined to be 600 mothers, 1800 overall. The final study sample included 1734 mothers from 90 villages, 3 districts, and 3 province; 1740 mothers were originally approved but 6 refused to participate.

### Procedure

Ethical approval was obtained from the Ethical Research Committee by the Faculty of Public Health, Indonesia University. Reconstra, a research firm from Jakarta, conducted the data collection. Signed informed consent was sought from each participant prior to the interview and participation of all women was voluntary and no compensation was provided. Survey data were collected using an electronic tablet by experienced interviewers and field coordinators. Each interviewer interviewed approximately 6 respondents per day and reported to field coordinators who then verified the responses and uploaded survey data daily. A data manager checked data and noted any errors. Data cleaning was done prior to analysis.

### Variables

#### Demographics

Demographic information was gathered from each participant and included mother’s age, mother’s highest level of education attained, and total household income (Multimedia Appendix 1). A measure of mothers’ use of technology was assessed by identifying respondents who used either a tablet, computer/laptop, or smartphone to access health services.

#### Knowledge and Behaviors Related to Handwashing and Defecation

Knowledge and behavior of respondents related to WASH were assessed by asking respondents to identify benefits of proper handwashing using soap (options include prevent germ transmission, reduce diarrhea, and prevent infection), 5 critical handwashing times (options include after defecation, after cleaning baby who defecated, before preparing meals, before eating meals, and before breastfeeding), when they used soap while handwashing in the previous 24 hours (options include after defecation, after cleaning baby who defecated, before preparing meals, before eating meals, and before breastfeeding), risks of open defecation (options include transmission of germs and diarrhea), media for disease transmission from stool to child (options include flies, water, and dirt), proper location for defecation (options include hygienic latrine), and where the respondent defecated at home (options include gooseneck toilet, squat toilet with or without floor, and pit latrine). Each response was coded as yes or no for the knowledge or behavior indicator. For example, each critical handwashing time identified was coded as a separate variable with a yes for those who identified each particular critical time. Proportions of respondents that reported each knowledge or behavior are provided.

Index indicators were created to summarize individual indicators described above. Indices created included proportion of respondents who could identify at least one correct benefit of handwashing with soap (yes vs no); the number of critical handwashing times identified; number of times soap was used while washing hands in the previous 24 hours (0-5); proportion of respondents who could identify at least one risk of open defecation (yes vs no); proportion of respondents who could identify at least one medium of stool to child disease transmission (yes vs no); number of correct places to defecate (0-5); and proportion of respondents who reported defecating in a gooseneck toilet, squat toilet with no floor, or squat toilet with floor, or discarded feces in a septic tank or a closed ground hole (yes vs no).

#### Use of Technology to Access Health Services and Impact on Knowledge and Behavior Related to Hygiene and Defecation

Respondents were asked whether they accessed health services using modern tools for communication, and if so, which technology they used (options include tablet, computer/laptop, or smartphone). An index indicator of access to health services was constructed by identifying respondents who used any of the 3 technologies to access health services (yes vs no). The relationship of using technology to access health services with knowledge and behavior indices related to hygiene and defecation was then assessed.

### Statistical Analysis

SAS version 9.4 (IBM) was used to calculate descriptive statistics. Regression models were used to assess the association between use of technology to access health services and each individual indicator of WASH-related knowledge and behavior. Adjusted models were also constructed to control for mother’s age, mother’s education, and total household income. These controls were added because of the association each variable has been shown to have on similar outcomes in previous studies [13,14,17,18]. Hence, the use of technology to access health services was examined with each WASH variable individually with regression analysis and then again with the standard controls using regression modeling. The health services technology variable was always used as the model predictor while the WASH variable was used as the outcome. Logistic regression was used to assess the association between health services technology and dichotomous WASH variables while linear regression was used when WASH variables were continuous. Logistic models included odds ratios (ORs) and 95% CI while linear regression models included point estimates and *P*-values.

## Results

There were a total of 1734 mothers with children under the age of 2 (Table 1). Most mothers had a primary school education, while few had tertiary education. Being unemployed or a housewife were the most common occupations. Other occupations included small trader, civil servant, and private employee. The mean total annual household income was €131.05 (US $160.04). Almost one-fifth of respondents have access to and used a phone, computer, or tablet to access health information and services.

**Table 1:** Participant demographics in Banyuasin, Kubu Raya, and Katingan in 2018 (N=1734).

Demographics		Values
Mean (SD) age		28.9 (6.30)
**Education, n (%)**		
	None	97 (5.59)
	Primary school	670 (38.64)
	Junior high school	423 (24.39)
	Senior high school	434 (25.03)
	Tertiary education	110 (6.34)
**Occupation, n (%)**		
	Unemployed or Housewife	1461 (84.26)
	Farmer	49 (2.83)
	Light traders/Shop owner	79 (4.56)
	Other	145 (8.36)
**Religion, n (%)**		
	Islam	1640 (94.58)
	Other	94 (5.42)
**Technology use, n (%)**		
	Phone	265 (15.28)
	Computer	14 (0.81)
	Tablet	16 (0.92)
	Any technology	276 (15.92)
Mean (SD) total household income (Euros)^a^		131.05 (116.17)^b^

^a^Indonesian Rupiah (official currency of Indonesia) was converted to Euros.

^b^US $160.04 (141.85).

In most cases of handwashing knowledge and behaviors, participants who used any technology to seek health information were better off than those who did not use technology (Table 2). For example, 91.7% (253/276) of participants who used technology knew at least one benefit of handwashing compared to 80.86% (1179/1458) of those who did not use technology. Further, 88.41% (244/276) of households who used technology for health reported using a hygienic location for defecation while 66.39% (968/1458) of households who did not use technology reported using a hygienic location.

**Table 2:** Knowledge and behaviors of handwashing and defecation by use of health technology among Indonesian women in Banyuasin, Kubu Raya, and Katingan in 2018.

Knowledge and behavior			Health technology use		*P*-value^a^
			Yes, n (%) N=276	No, n (%) N=1458	
**Handwashing**					
	Know at least one benefit of proper handwashing		253 (91.67)	1179 (80.86)	<.001
	**Know that handwashing can**				
		Prevent germ transmission	239 (86.59)	1076 (73.80)	<.001
		Decrease diarrhea	49 (17.75)	188 (12.89)	.03
		Prevent infection	19 (6.88)	98 (6.72)	.92
	**Know handwashing should occur**				
		After defecation	206 (74.64)	994 (68.18)	.03
		After cleaning baby/infant who defecated	108 (39.13)	638 (43.76)	.15
		Before preparing meals	147 (53.26)	758 (51.99)	.70
		Before eating meals	249 (90.22)	1199 (82.24)	.001
		Before breastfeeding/feeding child	135 (48.91)	594 (40.74)	.012
	Mean (SD) number of critical handwashing times participant identified (0-5)		3.1 (1.20)	2.87 (1.40)	.02^b^
	Mean (SD) number of times soap was used for handwashing since yesterday until today (0-5)		2.6 (1.30)	2.5 (1.30)	.25^b^
**Defecation**					
	Know the risks of open defecation		227 (82.25)	1079 (74.01)	.004
	Know about transmission of germs/*Escherichia coli* bacteria		176 (63.77)	775 (53.16)	<.001
	Know about causes of diarrhea		91 (32.97)	399 (27.37)	.06
	**Know mode of disease transmission from**				
		Stool	191 (69.20)	791 (54.25)	<.001
		Flies	149 (53.99)	562 (38.55)	<.001
		Water	59 (21.38)	258 (17.70)	.15
		Dirt	22 (7.97)	77 (5.28)	.08
	Know hygienic location for defecation		273 (98.91)	1377 (94.44)	.002
	Household uses gooseneck toilet or squat toilet with or without floor to defecate or septic tank or closed ground to discard feces		244 (88.41)	968 (66.39)	<.001

^a^Used chi-square test unless otherwise noted.

^b^Used *t* test.

Participants who used a health technology to access health services were more likely to know the benefits of proper handwashing as opposed to those who did not use health technology to access health services (OR 2.603, 95% CI 1.666-4.067; Table 3). After controlling for maternal age, maternal education level, and total household income, the use of technology to access health information and services was associated with knowledge of proper handwashing benefits (*P*=.004). Those who used technology to access health services were more likely to understand the media of disease transmission from stool to child.

**Table 3:** Use of technology to access health services and its impact on knowledge of hygiene and defecation in Banyuasin, Kubu Raya, and Katingan in 2018.^a^

Knowledge and behaviors					Unadjusted OR (95% CI)		Adjusted OR (95% CI)
**Handwashing**							
	Know at least one benefit of proper handwashing				2.60 (1.67-4.07)^b^		2.07 (1.26-3.41)^b^
	**Know that handwashing can**						
		Prevent germ transmission			2.29 (1.59-3.31)^b^		1.93 (1.28-2.91)^b^
		Decrease diarrhea			1.46 (1.03-2.06)^b^		1.17 (0.78-1.75)
		Prevent infection			1.03 (0.62-1.71)		0.69 (0.38-1.25)
	**Know handwashing should occur**						
		After defecation			1.37 (1.03-1.84)^b^		1.04 (0.75-1.44)
		After cleaning baby/infant who defecated			0.83 (0.64-1.08)		0.65 (0.05-0.88)^b^
		Before preparing meals			1.05 (0.81-1.36)		0.93 (0.70-1.25)
		Before eating meals			1.99 (1.31-3.03)^b^		1.66 (1.04-2.66)^b^
		Before breastfeeding/feeding child			1.39 (1.08-1.80)^b^		1.19 (0.89-1.59)
	Number of critical handwashing times participant identified (0-5)				0.19 (.0361)^c^		–0.01 (.8850)^d^
**Defecation**							
	Know the risks of open defecation				1.63 (1.17-2.26)^b^		1.21 (0.83-1.75)
	Know about transmission of germs/*Escherichia coli* bacteria				1.60 (1.23-2.09)^b^		1.51 (1.11-2.03)^b^
	Know about causes of diarrhea				1.31 (0.99-1.72)		0.96 (0.70-1.33)
	**Know mode of disease transmission from**						
			Stool	1.89 (1.44-2.50)^b^		1.57 (1.15-2.14)^b^	
			Flies	1.87 (1.44-2.42)^b^		1.52 (1.13-2.03)	
			Water	1.27 (0.92-1.74)		1.08 (0.75-1.55)	
			Dirt	1.55 (0.95-2.54)		1.38 (0.77-2.46)	
	Know hygienic location for defecation				5.35 (1.68-17.07)		2.22 (0.66-7.46)

^a^All adjusted models include maternal age, maternal education level, and total household income. Point estimates are derived from linear regression models while all odds ratios (ORs) are derived from logistic regression models.

^b^*P*<.05.

^c^Unadjusted point estimate (*P*-value).

^d^Adjusted point estimate (*P*-value).

Mothers with children under the age of 2 and who use technology to access health information and services have a greater chance of performing appropriate defecation behaviors (OR 3.85, 95% CI 2.62-5.66; Table 4). After adjusting for maternal age, maternal education level, and total household income, the use of technology to access health information and services was positively associated with using a gooseneck toilet or squat toilet with or without floor to defecate or a septic tank or closed ground to discard feces. The association of the use of technology to access health information and services with more hygienic handwashing behaviors was not statistically significant (*P*=.77).

**Table 4:** Use of technology to access health information and services and impact on behavior of hygiene and defecation in Banyuasin, Kubu Raya, and Katingan in 2018.^a^

Knowledge and behaviors			Value
**Handwashing**			
	**Number of times soap was used for handwashing since yesterday until today** **(0-5)**		
		Unadjusted point estimate (*P*-value)	0.10 (.249)
		Adjusted point estimate (*P*-value)	–0.03 (.771)
**Defecation**			
	**Household uses gooseneck toilet or squat toilet with or without floor to defecate or septic tank or closed ground to discard feces**		
		Unadjusted OR (95% CI)	3.86 (2.63-5.67)^b^
		Adjusted OR (95% CI)	2.32 (1.50-3.60)^b^

^a^All adjusted models include maternal age, maternal education level, and total household income. Point estimates are derived from linear regression models while all odds ratios (ORs) are derived from logistic regression models.

^b^*P*<.001.

## Discussion

### Principal Findings

The purpose of our study was to determine if using technology to access health information was associated with increased WASH knowledge and optimal behaviors regarding proper handwashing and defecation practices. After controlling for age, education, and income, the study findings show that mothers of children under the age of 2 who used technology to access health information and services were more likely to be aware of the benefits of proper handwashing and proper defecation practices. While the difference in sufficient handwashing behaviors between those who used technology to access health information and those who did not was nearly non-existent, the most significant finding of our study was that these mothers have a much higher likelihood of using appropriate defecation behaviors. This factor alone has the ability to reduce illness and could provide continual positive benefits for children and families. A campaign in India to decrease open defecation by promoting community latrine use concluded that communities that used latrines experienced reduced fecal contamination in the community and improved child arm circumference, weight, and height. Households also saved time [19]. Children from villages in India with community latrine coverage had significantly higher cognitive scores 10 years later [20] and children, especially girls, were less likely to drop out of school [21]. Another study in India showed that 30%-55% of the average differences in stunting between districts could be due to differences in open defecation [22].

Our study found that different media sources were not only associated with increased WASH knowledge but also associated with WASH behaviors. While this is the first finding of this type in Indonesia, the positive relationship between technology usage and WASH knowledge has been highlighted in previous research conducted in other countries. Previous research in rural Tanzania evaluated how media access impacts WASH knowledge and behaviors [10]. Media access in the Tanzanian study included listening to the radio, watching television, or having WhatsApp on a smartphone. Exposure to media was measured based on when the media was accessed. Participants could select from 5 options: today, yesterday, in the last week, in the last month, or more than a month ago. Like our study, results from the Tanzanian study showed a similar positive trend regarding technology access and increased handwashing. Specific findings from the Tanzanian study showed that participants who watched television had a positive correlation with increased WASH knowledge [10]. One potential reason for similar findings is that IMA–World Health sponsored the WASH media campaign in both countries. While the media campaign was adapted for cultural differences, the campaigns were likely similar and resulted in similar outcomes in both countries.

Mothers who used technology had a higher likelihood of knowing when it was appropriate and necessary to use proper handwashing but did not necessarily follow through with the appropriate behavior. This is valuable information for health agencies and service providers as it highlights where the implementation gaps are, and that increasing the use of technology may be a way to promote this information, at least for some topics. For example, many participants were able to identify at least one risk of open defecation, but less had specific knowledge about the mode of disease transmission. It is also surprising that technology use was associated with mothers’ knowledge of handwashing before eating a meal, but not with knowledge of handwashing before preparing a meal. It may be an indicator of a need for more emphasis on handwashing before meal preparation, whereas handwashing before eating a meal has been a consistent message. Another study that resulted in increased handwashing knowledge with no or minimal change in handwashing behavior was an interactive campaign in India. The interventions focused on using toilets and washing hands with soap. Those who participated in the campaign increased their knowledge about the benefits of handwashing by about half a standard deviation, but the change in intention to wash their hands was small [23]. Conversely, another study found that in India television advertising and SMS text messages using mobile phones increased the likelihood of mothers washing their hands [24]. While media has the potential to improve handwashing behaviors, barriers must be addressed. Three new television campaigns to increase handwashing among Australian Aboriginal communities were widely viewed and understood. However, 75% of participants indicated they would purchase more handwashing supplies if they were less expensive [25].

Our study results showed that more accurate knowledge regarding the probability of disease arising due to open defecation and proper defecation procedures could have an effect on behavior practices. Further research could be done as to how to allow more women to access health information through technology to improve the overall health and well-being of their families. This research might include exploring the technology needs and capacities of mothers. The percentage of mothers in this study that used technology to access health information was low. It is not clear if this is a function of cultural norms, income, or some other influence. Nevertheless, the promising association between mothers’ use of technology and knowledge is such that a study of this nature is warranted.

A study conducted in 7 of the 8 provinces of Kenya also found a positive correlation between accessing technology and handwashing behavior [26]. Participants were chosen from 7 of the 8 provinces in Kenya. Sources of media were divided into 2 categories: media ownership and media exposure occurring in the last month. Possible media sources in this study included, but were not limited to, television, radio, newspapers, and movies. Exposure to media was gauged by determining how many household items the participants owned or the amount of various media sources they were exposed to. Results indicated that each variable directly corresponded to increased handwashing practices. Additionally, both variables had a positive association with handwashing behavior that involved soap [26]. Data came from a cross-sectional survey conducted prior to a media and community-based handwashing program organized by the World Bank and the Kenyan Ministry of Health. The handwashing results from the Kenya study correspond with results from our study. Although defecation practices were included as sociodemographic characteristics, the paper did not address whether access to technology was associated with defecation behaviors. Further research is needed to determine whether other countries experience any relationship between technology and handwashing but not defecation behaviors.

### Study Limitations

A few limitations of our study should be considered when reviewing the results. First, our study did not utilize an asset index to measure poverty; rather, it used a total household income indicator. Second, the broader study, from which our data were derived, did not intend to examine the indicator for access to technology and how it relates to handwashing and defecation behaviors. These indicators were not a key focus of the broader study; however, they remain valuable to our research because of the relationship identified between access to technology and the practices of handwashing and defecation. However, the association between the use of technology to access health information and the increased WASH knowledge in mothers of children aged under 2 are imperative discoveries.

### Conclusions

In conclusion, the use of technology to access health information was associated with correct WASH knowledge, and with the use of safe methods of eliminating feces. However, using technology was not associated with an increase in the number of times of handwashing with soap. The findings of our study suggest several potential opportunities for furthering knowledge and creating behavior change as these relate to handwashing and defecation practices, thereby improving health.

## Appendix

Multimedia Appendix 1 Survey Instrument.

## Abbreviations

IMA: Interchurch Medical Assistance
NNCC: National Nutrition Communication Campaign
WASH: Water access, sanitation, and hygiene

## References

[1] World Health Organization (2013). Progress on Sanitation and Drinking Water.

[2] World Health Organization, United Nations Children's Fund (UNICEF) (2017). Safely Managed Drinking Water: Thematic Report on Drinking Water.

[3] Agustina R, Sari TP, Satroamidjojo S, Bovee-Oudenhoven IMJ, Feskens EJM, Kok FJ (2013). Association of food-hygiene practices and diarrhea prevalence among Indonesian young children from low socioeconomic urban areas. BMC Public Health.

[4] Liu L, Oza S, Hogan D, Perin J, Rudan I, Lawn JE, Cousens S, Mathers C, Black RE (2015). Global, regional, and national causes of child mortality in 2000-13, with projections to inform post-2015 priorities: an updated systematic analysis. Lancet.

[5] Rah JH, Cronin AA, Badgaiyan B, Aguayo VM, Coates S, Ahmed S (2015). Household sanitation and personal hygiene practices are associated with child stunting in rural India: a cross-sectional analysis of surveys. BMJ Open.

[6] Prendergast AJ, Humphrey JH (2014). The stunting syndrome in developing countries. Paediatr Int Child Health.

[7] Dewey K, Begum K (2011). Long-term consequences of stunting in early life. Matern Child Nutr.

[8] Depkes RI (2011). Badan penelitian dan pengembangan Kesehatan. Riset Kesehatan Dasar.

[9] Belew AK, Ali BM, Abebe Z, Dachew BA (2017). Dietary diversity and meal frequency among infant and young children: a community based study. Ital J Pediatr.

[10] Alexander CC, Shrestha S, Tounkara MD, Cooper S, Hunt L, Hoj TH, Dearden K, Kezakubi D, Atugonza V, West J, Crookston B, Hall C (2019). Media Access is Associated with Knowledge of Optimal Water, Sanitation and Hygiene Practices in Tanzania. Int J Environ Res Public Health.

[11] Fjeldsoe BS, Marshall AL, Miller YD (2009). Behavior change interventions delivered by mobile telephone short-message service. Am J Prev Med.

[12] Cormick G, Kim NA, Rodgers A, Gibbons L, Buekens PM, Belizán JM, Althabe F (2012). Interest of pregnant women in the use of SMS (short message service) text messages for the improvement of perinatal and postnatal care. Reprod Health.

[13] Wang N, Deng Z, Wen LM, Ding Y, He G (2019). Understanding the Use of Smartphone Apps for Health Information Among Pregnant Chinese Women: Mixed Methods Study. JMIR Mhealth Uhealth.

[14] Saronga NJ, Burrows T, Collins CE, Ashman AM, Rollo ME (2019). mHealth interventions targeting pregnancy intakes in low and lower-middle income countries: Systematic review. Matern Child Nutr.

[15] Handayani PW, Meigasari DA, Pinem AA, Hidayanto AN, Ayuningtyas D (2018). Critical success factors for mobile health implementation in Indonesia. Heliyon.

[16] Nabila M (2018). APJII: Penetrasi Pengguna Internet Indonesia Capai 143 Juta Orang. Dailysocial.

[17] White S, Thorseth AH, Dreibelbis R, Curtis V (2020). The determinants of handwashing behaviour in domestic settings: An integrative systematic review. Int J Hyg Environ Health.

[18] Bish A, Michie S (2010). Demographic and attitudinal determinants of protective behaviours during a pandemic: a review. Br J Health Psychol.

[19] Dickinson KL, Patil SR, Pattanayak SK, Poulos C, Yang J (2015). Nature’s Call: Impacts of Sanitation Choices in Orissa, India. Economic Development and Cultural Change.

[20] Orgill-Meyer J, Pattanayak SK (2020). Improved sanitation increases long-term cognitive test scores. World Development.

[21] Orgill-Meyer J (2020). Interaction of village and school latrines on educational outcomes in India Internet. Journal of Water, Sanitation and Hygiene for Development.

[22] Spears D, Ghosh A, Cumming O (2013). Open defecation and childhood stunting in India: an ecological analysis of new data from 112 districts. PLoS One.

[23] Seimetz E, Kumar S, Mosler H (2016). Effects of an awareness raising campaign on intention and behavioural determinants for handwashing. Health Educ Res.

[24] Tidwell JB, Gopalakrishnan A, Lovelady S, Sheth E, Unni A, Wright R, Ghosh S, Sidibe M (2019). Effect of Two Complementary Mass-Scale Media Interventions on Handwashing with Soap among Mothers. J Health Commun.

[25] McDonald E, Cunningham T, Slavin N (2015). Evaluating a handwashing with soap program in Australian remote Aboriginal communities: a pre and post intervention study design. BMC Public Health.

[26] Schmidt W, Aunger R, Coombes Y, Maina P, Matiko C, Biran A, Curtis V (2009). Determinants of handwashing practices in Kenya: the role of media exposure, poverty and infrastructure. Trop Med Int Health.

