# Supplementary material for: Use of Technology to Access Health Information/Services and Subsequent Association With WASH (Water Access, Sanitation, and Hygiene) Knowledge and Behaviors Among Women With Children Under 2 Years of Age in Indonesia: Cross-sectional Study
Source: JMIR Public Health Surveill. 2021 Jan 14;7(1):e19349. doi: 10.2196/19349 (PMC7843201; doi:10.2196/19349)
Supplement: Multimedia Appendix 1 [file publichealth_v7i1e19349_app1.pdf]

Respondent Code:……………………

"Midline Campaign Survey for National Communication Campaign – IMA WORLD HEALTH INDONESIA"

Questionnaire for Pregnant Women

CODE	QUESTIONS	CHOICES
qdate_of_interview	Interview date :	
qFC_name	Field Coordinator (FC) Name :	1. FC Dinda<br>2. FC Andi<br>3. FC Ester
qBYEnum_name	Name of Enumerator in Banyuasin :	1. Inas<br>2. Yuliana<br>3. Nurhayati<br>4. Desi<br>5. Devita<br>6. Cyntia<br>7. Mutia<br>8. Ike<br>9. Fitri<br>10. Nisa<br>11. Novia<br>12. Rani<br>13. Korlap Dinda
qKBEnum_name	Name of Enumerator in Kubu Raya :	1. Adi<br>2. Dhea<br>3. Dwi<br>4. Eva<br>5. Dillah<br>6. Imam<br>7. Lian<br>8. Okta<br>9. Ryan<br>10. Semah<br>11. Korlap Andi
qKTEnum_name	Name of Enumerator in Katingan :	1. Arbain<br>2. Barly<br>3. Adi<br>4. Dewi<br>5. Heni<br>6. Imron<br>7. Iskandar<br>8. Mindra<br>9. Meylan<br>10. Wani<br>11. Korlap Ester

Respondent Code:……………………

NOTES: **(*Read loudly and clearly)*** "My name is …, I'm currently assisting research which is being carried out by RECONSTRA and IMA WORLD HEALTH INDONESIA. This research is performed to assess the basic condition of nutrition and health in IMA's areas of working. One of the data collection methods in this research is by conducting a structured interview and you are selected as the interviewees. We hope you are willing to provide information which will be used by IMA to increase the quality of the program in the future.

Confidentiality and consent:

Before the interview begin, I'd like to ask for your consent to participate as the interviewee of this research. Please sign up this form as a proof of your willingness as an interviewee.

During the interview, I will inquire some subjects about nutrition and health. Your participation is voluntary. This interview will not cause any risk or negative impact to you, as every information that you provide will remain confidential and guaranteed by RECONSTRA and IMA WORLD HEALTH INDONESIA.

If there is any further question, you may directly ask to our supervisor.

Are you willing to be interviewed?

(1). Yes. (2). No → *Provide reason:* ……………………………………..

(signature)______________________(name)___________________ (date) _______________

We appreciate your participation.

A. RESPONDENT INFORMATION

CODE	No.	QUESTIONS	CHOICES
qdistrict	1	District	1. Banyuasin<br>2. Kubu Raya<br>3. Katingan
qsub_district1	2.1	[BANYUASIN] – Sub district	1. Banyuasin I<br>2. Banyuasin II<br>3. Suak Tapeh<br>4. Rantau Bayur
qsub_district2	2.2	[KUBU RAYA] – Sub district	1. Sungai kakap<br>2. Sungai raya<br>3. Terentang<br>4. Kubu
qsub_district3	2.3	[KATINGAN] – Sub district	1. Kamipang<br>2. Tasik payawan<br>3. Katingan kuala<br>4. Pulau malan
qvillage1	3.1	Banyuasin I – Village	1. Cinta manis lama<br>2. Mariana ilir<br>3. Mariana<br>4. Pematang palas<br>5. Perajen<br>6. Sungaigerong

			7. Sungairebo<br>8. Tirta sari
qvillage2	3.2	Banyuasin II – Village	1. Sungsang I<br>2. Sungsang II<br>3. Sungsang III<br>4. Sungsang IV<br>5. Telukpayo<br>6. Marga sungsang
qvillage3	3.3	Suak Tapeh – Village	1. Tanjung laut<br>2. Air senggiris<br>3. Biyuku<br>4. Meranti<br>5. Sedang<br>6. Sukaraja<br>7. Talang ipuh
qvillage4	3.4	Rantau Bayur – Village	1. Rantau bayur<br>2. Tebing abang<br>3. Lebung<br>4. Rantau harapan
qvillage6	3.5	Sungai Kakap – Village	1. Kalimas<br>2. Pal sembilan<br>3. Punggur kecil<br>4. Sungai belidak<br>5. Sungai rengas
qvillage7	3.6	Sungai Raya – Village	1. Kuala dua<br>2. Parit baru<br>3. Sungairaya<br>4. Tebang kacang<br>5. Telukkapuas
qvillage8	3.7	Terentang – Village	1. Sungai radak dua<br>2. Sungai radak Satu<br>3. Teluk bayur
qvillage9	3.8	Kubu – Village	1. Serut dua<br>2. Sungai selamat<br>3. Kubu<br>4. Pinang luar
qvillage10	3.9	Kamipang – Village	1. Baun bango<br>2. Galinggang<br>3. Jahanjang<br>4. Tampelas<br>5. Tumbang runen<br>6. Telaga

			7. Karuing
qvillage11	3.10	Tasik Payawan – Village	1. Hiyang bana<br>2. Luwuk kanan<br>3. Talingke<br>4. Tewang tampang
qvillage12	3.11	Katingan Kuala – Village	1. Makmur utama<br>2. Pangatan hulu<br>3. Bumi subur<br>4. Bangun jaya<br>5. Singam raya<br>6. Setia mulia<br>7. Kampung baru (katingan kuala)<br>8. Pagatan hilir<br>9. Bakung raya<br>10. Subur indah
qvillage13	3.12	Pulau Malan – Village	1. Trans SP 1<br>2. Tumbang tanjung<br>3. Manduing taheta<br>4. Tewang derayu<br>5. Buntut bali
qsub_village	4	Sub-village	
qhome_address	5	Address	
qname_posyandu	6	Posyandu Name	
qno_hp	7	Phone number	
qname_mother	8	**Mother's Name**	
qage_mother	9	Mother's Age	Years
qeducation_mother	10	Mother's Last Education	1] None<br>2] Primary School<br>3] Junior High School<br>4] Senior High School<br>5] Tertiary Education<br>99] Refused to answer
qoccupation_mother	11	Mother Occupation for the last 4 weeks	1] Unemployed/Housewife<br>2] Daily/labor worker<br>3] Industrial worker<br>4] Hunter<br>5] Farmer<br>6] Fisherman<br>7] Military/ police officer<br>8] Carpenter<br>9] Wholesalers<br>10] Light traders/shop owner<br>11] Civil servant/Government officer

			12] Car/ Motorcycle Driver<br>13] Private employee<br>97] Others:<br>____________________<br>99] Refused to answer
qreligion_mother	12	Mother's Religion	1] Islam<br>2] Catholic<br>3] Christian<br>4] Buddha<br>5] Hindu<br>6] Confucius<br>97] Others:<br>____________________<br>99] Refused to answer
qname_father	13	**Father's Name**	
qage_father	14	Father's Age	Years
qeducation_father	15	Father's Last Education	1] None<br>2] Primary School<br>3] Junior High School<br>4] Senior High School<br>5] Tertiary Education<br>99] Refused to answer
qoccupation_father	16	Father's Occupation for the last 4 weeks	1] Unemployed/Housewife<br>2] Daily/labor worker<br>3] Industrial worker<br>4] Hunter<br>5] Farmer<br>6] Fisherman<br>7] Military/ police officer<br>8] Carpenter<br>9] Wholesalers<br>10] Light traders/shop owner<br>11] Civil servant/Government officer<br>12] Car/ Motorcycle Driver<br>13] Private employee<br>97] Others:<br>____________________<br>99] Refused to answer
qincome_father	17	Do the father/head of the family who owns child under 2-year-old have income?	0] No<br>1] Yes<br>99] Refused to answer
qincome_father_permonth	18	If yes, how much do the father/head of the family earn per month?	99] If respondent does no know
qincome_mother	19	Do the mother who owns child under 2-year-old have income	0] No<br>1] Yes<br>99] Prefer not to anwer
qincome_mother_permonth	20	If yes, how much do the mother earn per month?	99] If respondent does not know

qincome_Other	21	Is there any other member of the family who have income?	0] No<br>1] Yes<br>99] Refused to answer
qincome_permonth	22	If yes, how much do they earn per month?	99] If respondent does not know
qreligion_father	23	Father's Religion	1] Islam<br>2] Catholic<br>3] Christian<br>4] Buddha<br>5] Hindu<br>6] Confucius<br>97] Others: _____<br>99] Refused to answer

Respondent Code:……………………

B. PRACTICE of Respondent about Key Message in National Nutrition Campaign

B1.Iron Folic Acid Tablets

CODE	NO	
qb1_1	1	During the pregnancy, is mother given or bought iron folic acid tablets (TTD)?<br><br>1 Yes<br>0 No (Continue to **B2.2**)<br>98 Not know or not sure<br>99 Refused to answer
qb1_2	2	Does mother consume iron folic acid tablets (TTD)?<br><br>1 Yes<br>0 No (Continue to **B2.2**)<br>98 Not know or not sure<br>99 Refused to answer
qb1_3	3	If yes, how often does the mother consume iron folic acid tablets (TTD)?<br>1. Every day<br>2. 2-3 times a week<br>3. irregular<br>98. Do not know<br>99. Refused to answer

---

B2. Practices of Meal Consumption

qb2_1	How many times per day does the mother eat their meal?<br>___________time(s)/day<br><br>99 Refused to answer
qb2_2	How many times per day does the mother consume her snack? _________time(s)/day<br><br>99. Refused to answer

B3. Morning Meal (Breakfast)

Now I'd like to ask you, what did you consume from yesterday (from waking up to night sleep)?

qb3_1	1	Staple food: such as corn, wheat, rice, grains, cassava, potato, etc.<br>1] Score 1<br>0] Score 0<br>99] Refused to answer
qb3_2	2	Nuts and beans: such as peanuts, green beans, walnuts, etc.<br>1] Score 1<br>0] Score 0<br>99] Refused to answer
qb3_3	3	Fruits containing Vitamin A and Vegetables: such as mango, papaya, orange, green veggies, pumpkin, and other fruits and vegetables, such as banana, pineapple, avocado, watermelon, tomato, cabbage, and eggplant.<br>1] Score 1<br>0] Score 0

		99] Refused to answer
qb3_4	4	Animal-based food containing iron: such as meat, chicken meat, liver, and egg, and animal-based food such as fish and others.<br>1] Score 1<br>0] Score 0<br>99] Refused to answer
qb3_5	5	Pregnancy Milk<br>1] Score 1<br>0] Score 0<br>99] Refused to answer

Snack : Morning

qb3_6	6	Fruits<br>1] Score 1<br>0] Score 0<br>97] Not relevant<br>99] Refused to answer
qb3_7	7	Cake<br>1] Score 1<br>0] Score 0<br>97] Not relevant<br>99] Refused to answer
qb3_8	8	Biscuit or cookies<br>1] Score 1<br>0] Score 0<br>97] Not relevant<br>99] Refused to answer
qb3_9	9	Pregnancy Milk<br>1] Score 1<br>0] Score 0<br>97] Not relevant<br>99] Refused to answer

B4. Lunch

qb4_1	1	Staple food: such as corn, wheat, rice, grains, cassava, potato, etc.<br>1] Score 1<br>0] Score 0<br>97] Not relevant<br>99] Refused to answer
qb4_2	2	Nuts and beans: such as peanuts, green beans, walnuts, etc.<br>1] Score 1<br>0] Score 0<br>97] Not relevant<br>99] Refused to answer

qb4_3	3	Fruits containing Vitamin A and Vegetables: such as mango, papaya, orange, green veggies, pumpkin, and other fruits and vegetables, such as banana, pineapple, avocado, watermelon, tomato, cabbage, and eggplant.<br>1] Score 1<br>0] Score 0<br>97] Not relevant<br>99] Refused to answer
qb4_4	4	Animal-based food containing iron: such as meat, chicken meat, liver, and egg, and animal-based food such as fish and others.<br>1] Score 1<br>0] Score 0<br>97] Not relevant<br>99] Refused to answer
qb4_5	5	Pregnancy Milk<br>1] Score 1<br>0] Score 0<br>97] Not relevant<br>99] Refused to answer

Snack : Afternoon

qb4_6	6	Fruits<br>1] Score 1<br>0] Score 0<br>97] Not relevant<br>99] Refused to answer
qb4_7	7	Cake<br>1] Score 1<br>0] Score 0<br>97] Not relevant<br>99] Refused to answer
qb4_8	8	Biscuit or cookies<br>1] Score 1<br>0] Score 0<br>97] Not relevant<br>99] Refused to answer
qb4_9	9	Pregnancy Milk<br>1] Score 1<br>0] Score 0<br>97] Not relevant<br>99] Refused to answer

B5. Diner

qb5_1	1	Staple food: such as corn, wheat, rice, grains, cassava, potato, etc.<br>1] Score 1<br>0] Score 0

		97] Not relevant<br>99] Refused to answer
qb5_2	2	Nuts and beans: such as peanuts, green beans, walnuts, etc.<br>1] Score 1<br>0] Score 0<br>97] Not relevant<br>99] Refused to answer
qb5_3	3	Fruits containing Vitamin A and Vegetables: such as mango, papaya, orange, green veggies, pumpkin, and other fruits and vegetables, such as banana, pineapple, avocado, watermelon, tomato, cabbage, and eggplant.<br>1] Score 1<br>0] Score 0<br>97] Not relevant<br>99] Refused to answer
qb5_4	4	Animal-based food containing iron: such as meat, chicken meat, liver, and egg, and animal-based food such as fish and others.<br>1] Score 1<br>0] Score 0<br>97] Not relevant<br>99] Refused to answer
qb5_5	5	Pregnancy Milk<br>1] Score 1<br>0] Score 0<br>97] Not relevant<br>99] Refused to answer

Makanan Selingan : Malam

qb5_6	6	Fruits<br>1] Score 1<br>0] Score 0<br>97] Not relevant<br>99] Refused to answer
qb5_7	7	Cake<br>1] Score 1<br>0] Score 0<br>97] Not relevant<br>99] Refused to answer
qb5_8	8	Biscuit/cookie<br>1] Score 1<br>0] Score 0<br>97] Not relevant<br>99] Refused to answer
qb5_10	9	Pregnancy Milk<br>1] Score 1<br>0] Score 0

		97] Not relevant<br>99] Refused to answer
B6. Changes in Eating Habit/Pattern		
qb6_1	1	During this pregnancy, did you experience dietary changes?<br>1] Yes<br>0] No (Continue to **B7**)<br>99] Refused to answer
qb6_2	2	In what ways did you experience dietary changes?<br>1] less than 2 portions<br>2] less than 1 portion<br>3] ½ portion more<br>4] 1 portion more<br>5] 2 portions more<br>97] others, please specify<br>99] Refused to answer

B7. Sanitation and Hygiene

	1	Do you have any soap in your house?<br>1] Yes<br>0] No (Continue to **B7.3**)<br>99] Prefer not to say
	2	Since yesterday until today, what do you usually use soap for?<br>(select all that apply)
qb7_21		Hand washing
qb7_22		Bath
qb7_23		Laundry
qb7_24		Dishwashing
qb7_299		Refused to answer
qothers_3		Other, please specify: _______________________
	2.1	Since yesterday until today, when do you use soap for hand washing? DO NOT READ THE OPTIONS<br>(select all that apply)
qb7_2_11		Before preparing meals
qb7_2_12		Before eating
qb7_2_13		Before feeding baby/child
qb7_2_14		After defecating
qb7_2_15		After defecating baby/child
qb7_2_199		Refused to answer

qothers_5		Other, please specify
qb7_3	3	Where do you and your family usually defecate?<br>1] Own toilet/latrine<br>2] Public toilet/latrine<br>3] Shared toilet/latrine<br>4] Does not have toilet/latrine (river, pond, or garden) [Continue to **B7.7**]<br>99] Refused to answer
qb7_4	4	What type of closet used at home?<br>1] Goose nect toilet<br>2] Squat toilet with no floor (*Cemplung/cubluk tanpa lantai)*<br>3] Squat toilet with floor (*Cemplung/cubluk dengan lantai*)<br>4] Pit toilet (*Plengsengan*)<br>99] Refused to answer
qb7_5	5	Where are the feces discarded?<br>1] Septic tank (SPAL) (Continue to **B7.6**)<br>2] Closed ground hole<br>3] Open ground hole<br>4] Pond/paddy field<br>5] River/lake/sea<br>6] Beach/field/garden<br>97] Othes, please specify<br>98] Do not know<br>99] Refused to answer
qb7_6	6	Has your septic tank ever been aspirated?<br>1] Yes<br>0] No<br>98] Do not know<br>99] Refused to answer
qb7_7	7	How far is the (drinking) water spring (for your household) to your closest feces disposal? [in meter]<br><br>99. Do not know

C. Respondent's KNOWLEDGE about the message in National Nutrition Communication Campaign (DO NOT READ THE OPTIONS)

C1. First 1000 days of Life

qc1_1	1	Do you know the most critical time for child to grow and develop?<br>1] Yes<br>0] No (Continue to **C1.4**)<br>99] Refused to answer
qc1_2	2	Can you mention the most critical time for child to grow and develop?<br><br>(select all that apply)
qc1_21		Pregnancy
qc1_22		Infant (during breastfeeding period)
qc1_23		Child age below 2 (during providing supplementary food period-ASI) From pregnancy to 2 years old/Child age below 2

qc1_298		Do not know
qothers_8		Other, please specify
	3	Can you mention attitude which encourages/enable the success of child's grow and development?<br>(select all that apply)
qc1_31		Consuming nutritious and iron folic acid during pregnancy
qc1_32		Providing exclusive breastfeeding for chil age 0-6 months
qc1_33		Providing various and balance supplementary food-breastfeeding for child age 6-24 months
qc1_398		Do not know
qothers_10		Others, please specify
qc1_399		Refused to answer
qc1_4	4	Have you heard about First 1000 days of life (*1000 Hari pertama kehidupan*)?<br>1] Yes<br>0] No<br>99] Refused to answer

C2. Stunting

	1	What would be the characteristics of undernourished child?<br>(select all that apply)
qc2_11		Short stature or delayed physical growth
qc2_12		Vulnerable to infection
qc2_13		Poor cognitive performance or delayed brain development
qc2_14		Easily gaining weight later at life (adult)
qc2_15		Skinny
qc2_198		Do not know
qc2_199		Refused to answer
qothers_12		Others, please specify
	2	What would be the consequences if child is undernutrition?<br>(select all that apply)
qc2_21		Delayed brain development
qc2_22		Delayed physical development
qc2_23		Lack of achievement
qc2_24		Vulnerable to infection or sickness
qc2_25		Easily gaining weight later at life (adult)
qc2_26		At risk of having degenerative disease

qc2_298		Do not know
qc2_299		Refused to answer
qothers_14		Others, please specify
qc2_3	3	Why can child be malnourished?<br>(select all that apply)
qc2_31		They have been undernutrition in their first 1000 days of life (since fetus to child age 2)
qc2_32		Lack of nutrition because of inappropriate parenting (Providing meal which is not corresponding with nutrition need and intake, it usually occurs to child age 6 months to 2 years)
qc2_33		Bacterial infection/germs because of unhealthy environment because of bad sanitation/open defecation which causes sickness
qc2_34		Open defecation
qc2_35		No-soap handwashing
qc2_36		Not consuming iron folic acid tablets
qc2_37		Lack of iron rich food (Chicken liver, egg, fish)
qc2_38		Mother is not actively feeding the child
qc2_398		Do not know
qothers_16		Others, please specify
qc2_4	4	Is malnutrition preventable?<br>1] Yes<br>0] No (Continue to **C2.6**)<br>98] Do not know<br>99] Refused to answer
	5	How do you prevent undernutrition?<br>(select all that apply)
qc2_51		Ensure good health and nutrition in the 1st 1000 days of life
qc2_52		Consume balance diet during pregnancy
qc2_53		Exclusive breastfeeding in the 1st 6 months of life
qc2_54		Proper complementary feeding for child age 6 months to 2 years
qc2_55		Prolonged breastfeeding up to 2 years of age
qc2_56		Children live in health and clean environment
qc2_57		Use a proper latrine
qc2_58		Consume iron folic acid tablets
qc2_59		Consume iron rich food (chicken liver, egg, fish)
qc2_510		Mother is actively feeding her child
qc2_511		Handwashing using soap
qc2_598		Do not know

qc2_599		Refused to answer
qothers_18		Others, please specify
qc2_6	6	Have you heard about STUNTING?<br>1] Yes<br>0] No<br>99] Refused to answer

C3. Iron Folic Acid Tablets

qc2_6	1	Have you ever heard or seen iron folic acid tablets (TTD) like this? (*show picture*)<br>1] Yes<br>0] No (Continue to **C4**)
qc3_1	2	If yes, when a pregnant woman should consume iron folic acid tablets /TTD?<br>1 – Consume 1 tablet every day during pregnancy<br>98 – Do not know<br>99 – Refused to answer
	3	Please mention the benefit of consuming iron folic acid tablets for pregnant women?<br>(select all that apply)
qc3_31		Not easily get headache, fatigue
qc3_32		Prevent anemia
qc3_33		Healthy fetus
qc3_34		Smooth labor
qc3_398		Do not know
qc3_399		Refused to answer
qothers_21		Others, please specify
	4	Please mention side effects of consuming iron folic acid tablets?<br>(select all that apply)
qc3_41		Nausea/gastric irritation
qc3_42		Vomit
qc3_43		Sometimes got diarrhea
qc3_44		Constipation
qc3_45		There is no side effect
qc3_498		Do not know

qc3_499		Refused to answer
qothers_23		Others, please specify
	5	How to consume iron folic acid tablets properly?<br>(select all that apply)
qc3_51		Consume with plain water
qc3_52		Consume with fruit juice or vitamin C
qc3_53		Consume right before sleeping
qc3_598		Do not know
qothers_25		Others, please specify

C4. IRON RICH FOOD (Chicken liver, egg, fish) as sources of iron

qc4	C4	Have you ever heard or known about iron rich food?<br>1] Yes<br>0] No (Continue to **C4.4**)<br>99] Refused to answer
	1	What do you think is the importance of consuming iron rich food?<br>(select all that apply)
qc4_11		Formation of red blood cell
qc4_12		To be healthy
qc4_13		To prevent anemia
qc4_198		Do not know
qothers_27		Others, please specify
	2	What would be the consequences of iron deficiency during pregnancy?<br>(select all that apply)
qc4_21		Cause anemia during pregnancy
qc4_22		At a risk of having premature birth, congenital defect, or low birth weight
qc4_23		Increase the risk of infant mortality
qc4_298		Do not know
qc4_299		Refused to answer
qothers_29		Others, please specify
	3	What are the resources of iron rich food?<br>(select all that apply)

qc4_31		Chicken liver
qc4_32		Egg
qc4_33		Fish
qc4_34		Meat
qc4_35		Green leafy vegetables
qc4_36		Tofu/Tempe/soy-based food
qc4_37		Other beans or nuts
qc4_398		Do not know
qothers_31		Others, please specify
qc4_4	4	Have you ever heard **about ATIKA?**<br>1] Yes<br>0] No (Continue to **C5**)<br>99] Refused to answer
qc4_4_1	4.1	If yes, what does the ATIKA stand for?<br>1] Chicken liver, Egg, and Fish<br>2] Other than Chicken liver, Egg, and Fish<br>99] Refused to answer

C5. Active Provision of (Supplementary) Meal

qc5_1	1	How many times should the child age below 2 be given meal/diet?<br>1 — 3X a day<br>98 — Do not know<br>97 — Others, please specify<br>99 — Refused to answer
qc5_2	2	How many times should the child age below 2 be given snack?<br>1 — 2X a day<br>98 — Do not know<br>97 — Others, please specify<br>99 — Refused to answer
	3	What would be the nutritious snack for your child?<br>(select all that apply)<br>1 — Fruits<br>2 — Cakes<br>3 — Biscuits<br>98 — Do not know<br>97 — Others, please specify<br>99 — Refused to answer
qc5_31		Fruits
qc5_32		Cakes

qc5_33		Biscuits
qc5_398		Do not know
qothers_35		Others, please specify
	4	What does it mean by nutritious diet for child age below 2?<br>(select all that apply)<br><br>1 Food containing '4 stars'<br>2 Staple food<br>3 Vegetables and Fruits<br>4 Beans<br>5 Animal food sources<br>98 Do not know<br>97 Others, please specify<br>99 Refused to answer
qc5_41		Food containing '4 stars'
qc5_42		Staple food
qc5_43		Vegetables and Fruits
qc5_44		Beans
qc5_45		Animal protein
qc5_498		Do not know
qc5_499		Refused to answer
qothers_37		Others, please specify
qc5_5	5	How do you encourage/enable your child to eat?<br>1 Asking them to play<br>98 Do not know<br>97 Others, please specify<br>99 Refused to answer

C6. Usage of Latrine

qc6_1	1	Do you know where you should defecate?<br>1 Hygienic latrine (closet and septic tank)<br>2 Unhygienic latrine<br>98 Do not know<br>97 Others, please specify<br>99 Refused to answer
	2	Can you mention the risk of open defecation?<br>(select all that apply)
qc6_21		Transmission of germs / E-Coli bacteria
qc6_22		Diarrhea
qc6_298		Do not know
qc6_299		Refused to answer

qothers_41		Others, please specify
	3	Do you know what 'media' that germs from feces can be transmitted to child?<br>(select all that apply)
qc6_31		Flies
qc6_32		Water
qc6_33		Dirt
qc6_398		Do not know
qc6_399		Refused to answer
qothers_43		Others, please specify

C7. Proper Handwashing

	1	Please mention the benefits of proper handwashing using soap (*Cuci Tangan Pakai Sabun* (CTPS))?<br>(select all that apply)
qc7_11		To prevent germs transmission
qc7_12		To decrease diarrhea
qc7_13		To prevent worm infection, eyes and skin infection
qc7_198		Do not know
qc7_199		Refused to answer
qothers_45		Others, please specify
	2	Please mention 5 critical times to handwash using soap<br>(select all that apply)
qc7_21		After defecation
qc7_22		After cleaning baby/infant who defecate
qc7_23		Before preparing meals
qc7_24		Before meals
qc7_25		Before breastfeeding/feeding child
qc7_298		Do not know
qc7_299		Refused to answer
qothers_47		Others, please specify

D. *ATTITUDE and INTENTTION about message in National Nutrition Communication Campaign (BADUTA)*

% respondent who reported relevance of NNCC message with needs
% respondent who intends to perform main key message of NNCC.

Respondent Code:……………………

	No	Statement	
qd1	1	d1.In my opinion, the 1st 1000 days of life is an important period for children	Disagree 1 2 3 4 Agree
qd2	2	d2.when I was pregnant, I DO NOT HAVE to consume 1 iron tablets daily during pregnancy	Disagree 1 2 3 4 Agree
qd3	3	d3.If I am pregnant, I felt the people who mattered to me encouraged me to consume 1 iron tablets daily during pregnancy	Disagree 1 2 3 4 Agree
qd4	4	d4.Consume one iron tablets daily during pregnancy is easy to do	Disagree 1 2 3 4 Agree
qd5	5	d5. If I am pregnant, I will consume one iron tablets daily during pregnancy	Disagree 1 2 3 4 Agree
qd6	6	d6. Eat one serving of Chicken's liver, Egg or Fish alternately every day during pregnancy, is needed by pregnant women to meet the needs of iron	Disagree 1 2 3 4 Agree
qd7	7	d7.Eat one serving of Chicken's liver, Egg or Fish alternately every day during pregnancy is easy to do	Disagree 1 2 3 4 Agree
qd8	8	d8. I felt the people who mattered to me NOT encouraged me to Eat one serving of Chicken's liver, Egg or Fish alternately every day during pregnancy	Disagree 1 2 3 4 Agree
qd9	9	d9.If I am pregnant, I am lazy to eat one serving of Chicken's liver, Egg or Fish alternately every day during pregnancy	Disagree 1 2 3 4 Agree
qd14	14	d14. Defecating in a healthy latrine is useful for preventing germs that cause diarrhea from spreading	Disagree 1 2 3 4 Agree
qd15	15	d15. Defecation in a healthy latrine is difficult	Disagree 1 2 3 4 Agree
qd16	16	d16. I felt the people who mattered to me NOT encouraged me to defecate in healthy latrine	Disagree 1 2 3 4 Agree
qd17	17	d17. I will defecate using healthy latrine	Disagree 1 2 3 4 Agree
qd18	18	d18. Washing hands with soap is useful to prevent diarrhea	Disagree 1 2 3 4 Agree
qd19	19	d19. It is DIFFICULT to wash hands with soap after defecating, after wiping the child's stool, before	Disagree 1 2 3 4 Agree

		preparing a meal, before eating, and before feeding the child	
qd20	20	d20. I felt the people who mattered to me NOT encouraged me to wash hands with soap after defecating, after wiping the child's stool, before preparing a meal, before eating, and before feeding the child	Disagree 1 2 3 4 Agree
qd21	21	d21. I will wash my hands with soap after defecating, after wiping the child's stool, before preparing a meal, before eating, and before feeding the child	Disagree 1 2 3 4 Agree
qd22	22	d22. Giving children nutritious food 3 times a day and fruit snacks 2 times a day is beneficial so that children will grow smart and tall	Disagree 1 2 3 4 Agree
qd23	23	d23. Giving children nutritious food 3 times a day and fruit snacks 2 times a day is DIFFICULT	Disagree 1 2 3 4 Agree
qd24	24	d24. I felt the people who mattered to me NOT encouraged me to give children nutritious food 3 times a day and fruit snacks 2 times a day	Disagree 1 2 3 4 Agree
qd25	25	d25. I will feed my child nutritious food 3 times a day and fruit snacks 2 times a day	Disagree 1 2 3 4 Agree

F. Level of Exposure to National Nutrition Communication Campaign

F.A. Level of Exposure of National Nutrition Communication Campaign: MASS Media/ Television

	No	TV Commercial TIPS to persuade child to eat	Message related to topic that you remember?*
qf1	1	From last year until this month, have you ever watched this commercial TV? 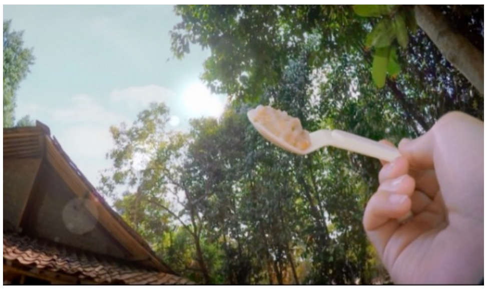	1] Yes<br>0] No<br>99] Refused to answer
	1.1	What is the message of the TV commercial that you remember?*<br><br>* (DO NOT READ THE OPTIONS, SELECT ALL OPTIONS THAT APPLY, IF PARTLY ANWERS, PLEASE CORRESPOND WITH AVAILABLE OPTIONS)	

qf1_11		Child should be invited to chat and play	
qf1_12		Supplementary food – breastfeeding starts on 6 months	
qf1_13		Have a proper meal 3 times a day	
qf1_14		Eat snack 2 times a day	
qf1_15		In order to increase child's physical and brain development	
qf1_16		Prolong breastfeeding to 2 years old	
qf1_17		Further information? Go to *Posyandu*	
qf1_18		Smart and high achieving child	
qf1_198		Do not know/not remembering	
qf1_199		Refused to answer	
qothers_49		Others, please specify	
		TV Commercial about Latrine	
qf2	2	From last year until this month, have you ever watched this commercial TV?<br>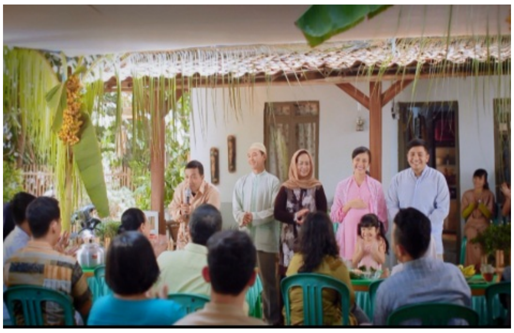	1] Yes<br>0] No<br>99] Refused to answer
	2.1	What is the message of the TV commercial that you remember?*<br><br>* (DO NOT READ THE OPTIONS, SELECT ALL OPTIONS THAT APPLY, IF PARTLY ANWERS, PLEASE CORRESPOND WITH AVAILABLE OPTIONS)<br>1. Building latrine is not expensive<br>2. Using latrine is more comfortable<br>3. Complete it with a septic tank<br>4. Smart and high achieving child starts from clean environment<br>5. Handwash using soap<br>98. Do not know/not remembering<br>97.<br>95. Not relevance<br>99. Refused to answer	
qf2_11		Building latrine is not expensive	
qf2_12		Using latrine is more comfortable	
qf2_13		Complete it with a septic tank	

qf2_14		Smart and high achieving child starts from clean environment	
qf2_15		Handwash using soap	
qf2_198		Do not know/not remembering	
qothers_51		Others, please specify	
		TV Commercial about stunting	
qf3	3	From last year until this month, have you ever watched this commercial TV?<br>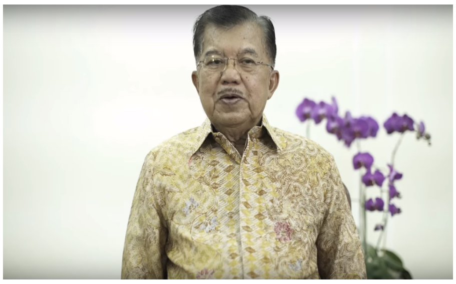	1] Yes<br>0] No<br>99] Refused to answer
	3.1	What is the message of the TV commercial that you remember?*<br>* (DO NOT READ THE OPTIONS, SELECT ALL OPTIONS THAT APPLY, IF PARTLY ANWERS, PLEASE CORRESPOND WITH AVAILABLE OPTIONS)	
qf3_11		Prevent stunting	
qf3_12		Preventing stunting is important	
qf3_198		Do not know/not remembering	
qf3_199		Refused to answer	
qothers_53		Others, please specify	
		Iklan Tv Stunting	
qf4	4	From last year until this month, have you ever watched this commercial TV?<br>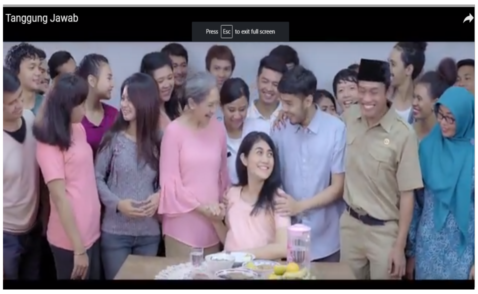	1] Yes<br>0] No<br>99] Refused to answer
	4.1	What is the message of the TV commercial that you remember?*	

		* (DO NOT READ THE OPTIONS, SELECT ALL OPTIONS THAT APPLY, IF PARTLY ANWERS, PLEASE CORRESPOND WITH AVAILABLE OPTIONS)
qf4_11		Mother is not alone in rising child
qf4_12		Cooperation with stakeholders in preventing stunting
qf4_13		Importance in preventing stunting
qf4_198		Do not know/not remembering
qf4_199		Refused to answer
qothers_55		Others, please specify
qf4_2	4.2	From all the videos that you have watched, which one is the most relatable with your needs?<br>1] Persuading child to eat<br>2] Usage of Latrine<br>3] Healthy child, we are all succeeded<br>4] Joint responsibility<br>99] Refused to answer

F.B. Level of Exposure and Engagement in NNCC: NNCC in community (inquired on behalf of family)

		PLEASE EXPLAINS THE DEFINITION OF "COUNSELLING" TO RESPONDENT BEFORE INQUIRING THESE QUESTIONS<br><br>Definition of Counselling:<br>Discussion between a counsellor (health workers or cadres) with mother or father in order to identify and solve nutrition and health issue that is currently experienced by pregnant mother or breastfeeding mother, or mother with infant (*below 5*).
qf5	5	Do your family ever attend counseling?<br>[1] Yes.<br>[2] Never (Continue to **F10)**<br>[98] Do not know<br>[99] Refused to answer
qf5_1	5.1	How many times in the last 5 months (August 2017 – Jan 2018)?________________________times
	6	Who provided counselling?<br>**(DO NOT READ OPTIONS, CIRCLE ALL THAT APPLY)**<br>_______________________________________
qf61		Cadre
qf62		Midwife
qf63		Doctor

qf64		Nurse
qf65		Nutritionist
qothers_57		Others, please specify
	7	What was the topic of counselling?<br>**(Do not read the options, circle all answers that apply)**
qf71		Active feeding
qf72		Hygienic environment: Usage of latrine and Handwashing with soap (*Cuci tangan pakai sabun* (CTPS))
qf73		Importance of Iron Rich Food and IFA tablets
qf799		Refused to answer
qothers_59		Others, please specify____
qf8	8	What was the benefit of counselling?<br><br>[99] Refused to answer
qf9	9	What's your suggestion from you to improve the counselling services?<br><br>[99] Refused to answer
		EXPLAINS DEFINITION OF "MOTHER CLASS" TO RESPONDENT BEFORE INQUIRING THESE QUESTIONS<br><br>Definition of mother class or support group for mother: peer-groups, comprises of pregnant mothers, mother with child age below 2, father, mother/father-in-law who meet regularly (minimum 1 time per month) to share experience, discuss, and give support for mother and child's health primarily related to pregnancy, breastfeeding, and nutrition which was facilitated by midwife/Posyandu cadre/Kesling/nutritionist/motivator/district facilitator. **Example of Mother Class: Class for pregnant mother, class for mother with infants/child age below 2**
qf10	10	Have your family member participated in mothers' class or support group for mother?<br>[1] Yes<br>[2] No (continue to **F.15**)<br>[98] Do not know<br>[99] Refused to answer
qf10_1	10.1	How many times did you participate (during Sept – Oct2016)?<br>________________________times<br><br>[99] Refused to answer, Do not know/Not remembering
	11	Who facilitated the class?<br>**(Do not read the options, circle all answers that apply)**
qf111		Cadre
qf112		Midwife
qf113		Doctor

qf114		Nurse
qf115		Nutritionist
qf1199		Refused to answer
qlainnya38		Others, please specify____
	12	What topic did you/family member participate? **(Do not read the options, circle all answers that apply)**
qf121		1st 1000 days of life is a golden period to boost health, smart, and high achieving child
qf122		Defection in Toilet: Defection, Hygienic Latrine in order to prevent germ transmission.
qf123		Handwashing using soap
qf124		IFA Tablets
qf125		Active feeding
qf126		Iron rich food
qf1299		Refused to answer
qcopy31oflainnya6		Others, please specify____
qf13	13	What was the benefit of the class?<br><br>[99] Refused to answer
qf14	14	What is your suggestion to improve the class?<br><br>[99] Refused to answer
	15	What are other activities in your community that strongly relates to the health which have been participated by you/your family member (other than counselling and mother class/support group class for mother)? **(Do not read the options, circle all answers that apply)**
qf151		Growth and development monitoring in Posyandu
qf152		Meeting for fathers
qf153		Pregnancy examination
qf154		Never participate in any activities
qf1598		Do not know
qf1599		Refused to answer
qcopy32oflainnya6		Others, please specify

F.C. Level of Exposure and Engagement in NNCC: Communication, Information and Education Materials (poster, banner and brochure, dll)

Respondent Code:……………………

	No	Materials	Have you ever seen/known it?
qf18	18	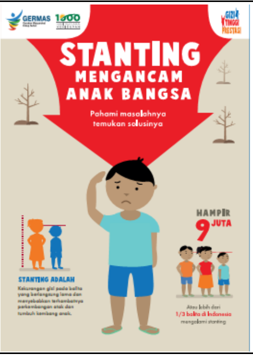	1] Yes<br>0] No<br>99] Refused to answer
qf19	19	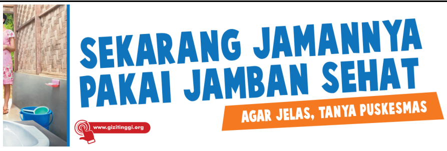	1] Yes<br>0] No<br>99] Refused to answer
qf20	20	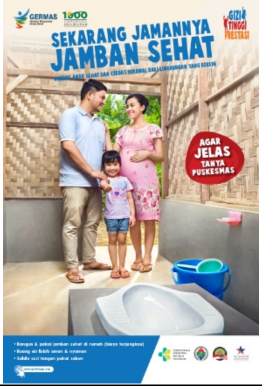	1] Yes<br>0] No<br>99] Refused to answer
qf21	21	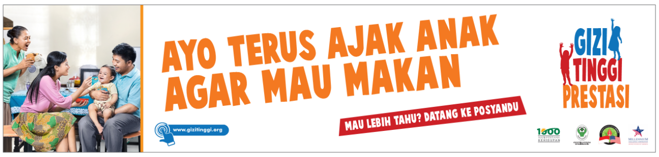	1] Yes<br>0] No<br>99] Refused to answer
qf22	22	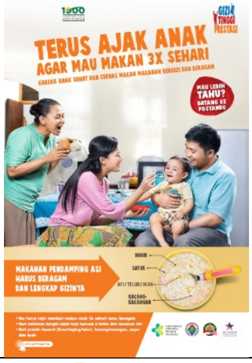	1] Yes<br>0] No<br>99] Refused to answer
qf23	23	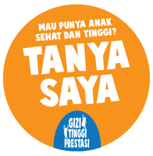	1] Yes<br>0] No<br>99] Refused to answer

qf24	24	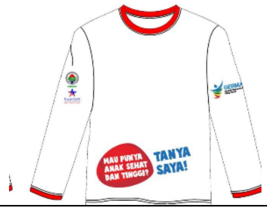	1] Yes<br>0] No<br>99] Refused to answer

Have you ever participated in these following activities in Posyandu or in other meetings since last October?

	**No**	**Activities**
qf25	25	Have you ever participated in singing: *Lagu tinggi Prestasi (*High Achievement Song)<br>1] Yes<br>0] No<br>99] Refused to answer
qf25_1	25.1	What is the key message of the song?<br>[1] 1st 1000 days of life is a golden period to support health, smart, and high-achieving child<br>[97] Others, please specify: ____<br>[98] Do not know/not remembering<br>[99] Refused to answer
qf26	26	Have you ever played: "'Bomb/Booby feces trap game' (*Bom/ ranjau Tai)*?<br>1] Yes<br>0] No<br>99] Refused to answer
qf26_1	26.1	What is the key message of the game?<br>[1] Defecation in latrine to prevent diarrhea causes to spread to child<br>[97] Others, please specify: ____<br>[98] Do not know/Not remembering<br>[99] Refused to answer
qf27	27	Have you ever: role play of cooking mothers, one who handwashes before cooking and one who does not handwash before cooking<br>1] Yes<br>0] No<br>99] Refused to answer
qf27_1	27.1	What is the key message of the activity?<br>[1] Handwash with soap<br>[97] Others, please specify: ____<br>[98] Do not know/Not remembering<br>[99] Refused to answer
qf28	28	Have you ever participated in 'Moving the stone games' either individually or in groups?<br>1] Yes<br>0] No<br>99] Refused to answer
qf28_1	28.1	What is the key message of the activity?<br>[1] Iron Folic Acid Tablets<br>[97] Others, please specify: ____<br>[98] Do not know/Not remembering<br>[99] Refused to answer

Respondent Code:……………………

qf29	29	Have you ever discussed and role played feeding food for child?<br>1] Yes<br>0] No<br>99] Refused to answer
qf29_1	29.1	What is the key message of the activity?<br>[1] Active feeding<br>[97] Others, please specify: ________________________________<br>[98] Do not know/Not remembering<br>[99] Refused to answer
qf30	30	Have you ever participated in measuring rice?<br>1] Yes<br>0] No<br>99] Refused to answer
qf30_1	30.1	What is the key message of the activity?<br>[1] Iron Rich Food as sources of iron<br>[97] Others, please specify: ________________________________<br>[98] Do not know/Not remembering<br>[99] Refused to answer

F.D. Level of Exposure and Engagement in NNCC: Communication, Information and Education Materials (Video)

	No	**Have you ever watched the scene?**	**Answers**
qf31	31	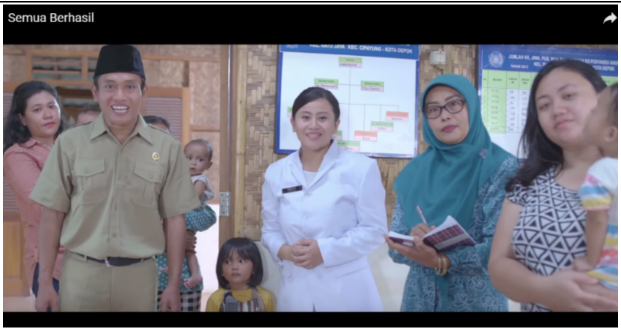<br>Have you ever watched the scene?	1] Yes<br>0] No<br>99] Refused to answer
qf32	32	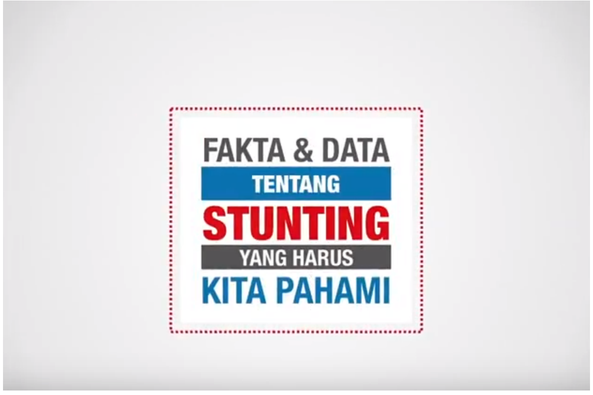<br>Have you ever watched the scene?	1] Yes<br>0] No<br>99] Refused to answer

qf33	33	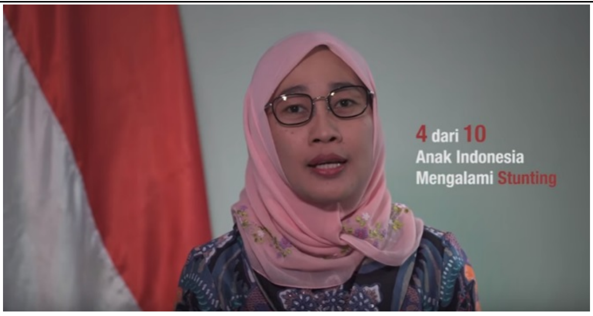<br><br>Have you ever watched the scene?	1] Yes<br>0] No<br>99] Refused to answer
qf34	34	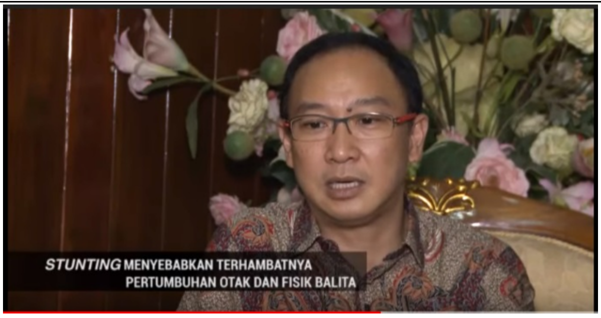<br><br>Have you ever watched the scene?	1] Yes<br>0] No<br>99] Refused to answer
qf35	35	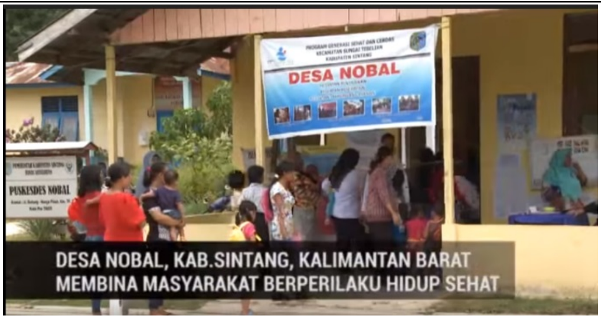<br><br>Have you ever watched the scene?	1] Yes<br>0] No<br>99] Refused to answer
qf36	36	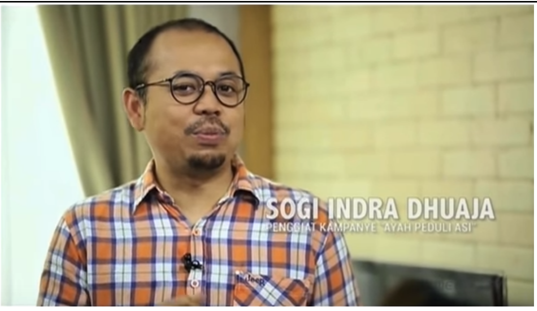<br><br>Have you ever watched the scene?	1] Yes<br>0] No<br>99] Refused to answer

Respondent Code:……………………

qf37	37	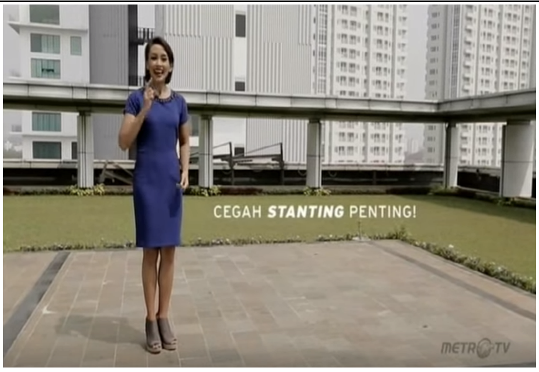 Have you ever watched the scene?	1] Yes<br>0] No<br>99] Refused to answer
qf38	38	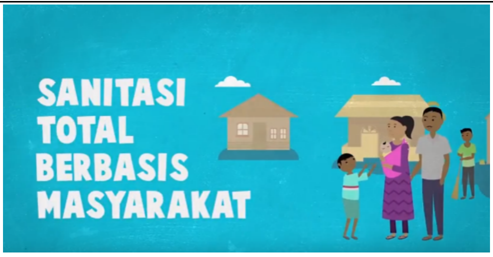 Have you ever watched the scene?	1] Yes<br>0] No<br>99] Refused to answer
qf39	39	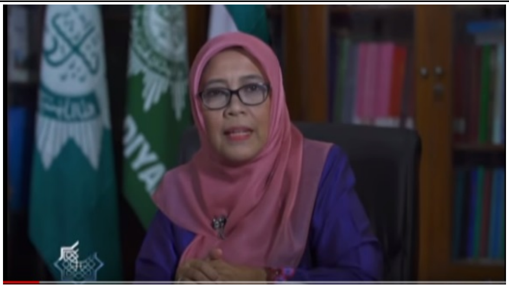 Have you ever watched the scene?	1] Yes<br>0] No<br>99] Refused to answer
qf40	40	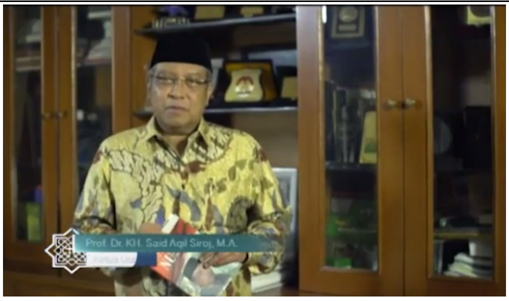 Have you ever watched the scene?	1] Yes<br>0] No<br>99] Refused to answer

<table>
<tr><td>qf41</td><td>41</td><td>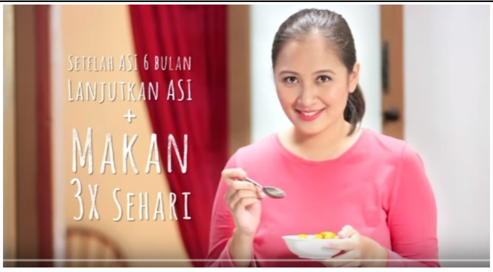

Have you ever watched the scene?</td><td>1] Yes
0] No
99] Refused to answer</td></tr>
</table>

F.E. Level of Exposure and Engagement in NNCC: Communication, Information and Education Materials (Social Media)

<table>
<tr><td>qf42</td><td>42</td><td>Have you ever visited “Gizi Tinggi Prestasi” website? (Show picture)
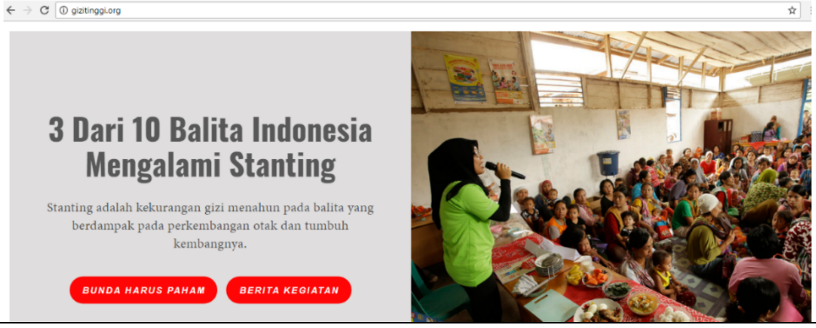
</td><td>1] Yes
0] No

99] Refused to answer</td></tr>
<tr><td>qf43</td><td>43</td><td>Have you ever visited “Gizi Tinggi Prestasi” twitter? (Show picture)
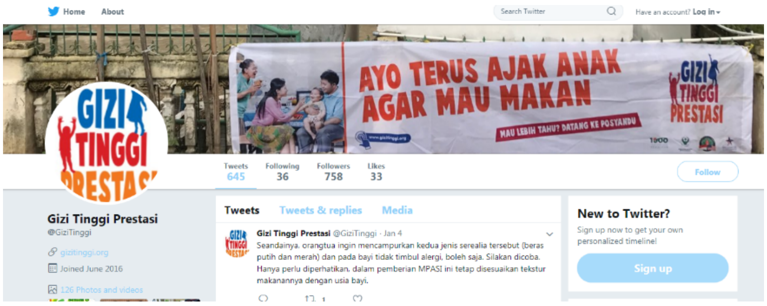
</td><td>1] Yes
0] No

99] Refused to answer</td></tr>
<tr><td>qf44</td><td>44</td><td>Have you ever visited “Gizi Tinggi Prestasi” instagram? (Show picture)</td><td>1] Yes
0] No

99] Refused to answer</td></tr>
</table>

		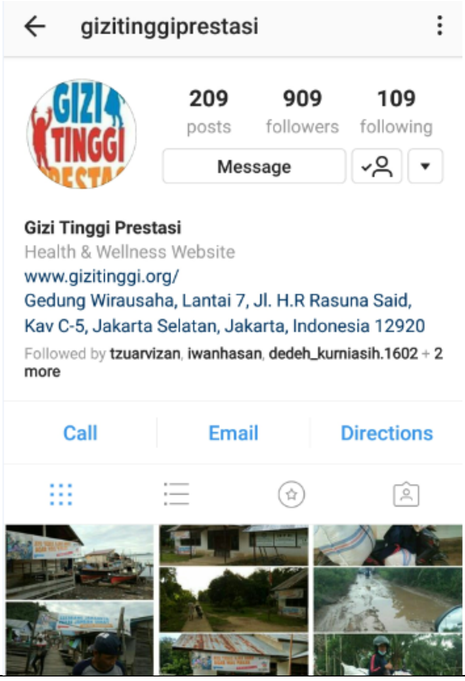	
qf45	45	Have you ever visited “Gizi Tinggi Prestasi” facebook page? (Show picture)<br>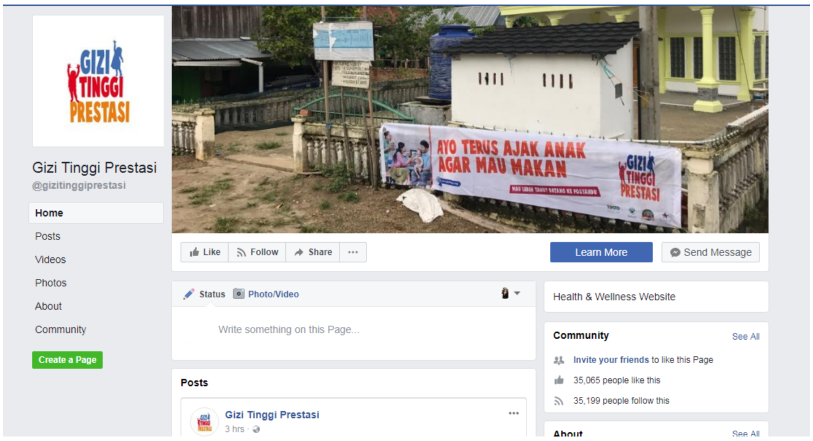	1] Yes<br>0] No<br><br>99] Refused to answer

G. *SOSIO ECONOMIC OF RESPONDENT*

	**G1**	**Receiving Poverty Reduction/Aid Program**
qg1_1	1	At the moment, do your family receive Family Hope Program (*Program Keluarga Harapan* (PKH))?<br>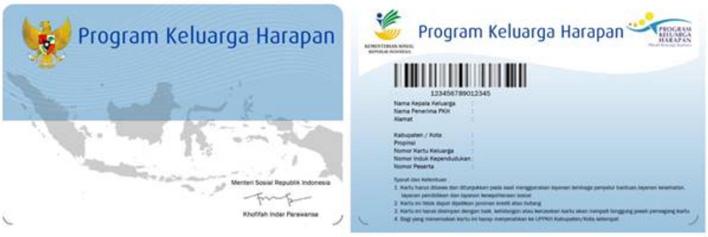<br>1] Yes<br>0] No

		99] Refused to answer
qg1_2	2	At the moment, do your family receive Subsidized Rice Program (*Program Beras Sejahtera*/ RASTRA/RASKIN)?<br><br>1] Yes<br>0] No<br>99] Refused to answer
	**G.2**	**Drinking sources**
qg2_<br>1	1	What is the main drinking sources for the family?<br>1. Water tap (PDAM, PAM, BPAM / HPAM / Water Committee)<br>2. Boreholes<br>3. Protected Dug wells<br>4. Unprotected Dug wells<br>5. Protected springs<br>6. Unprotected springs<br>7. Rainwater<br>8. Carts / water tank trucks<br>9. Refill water<br>10. Mineral bottled water<br>11. Surface water (river / pond / lake / sea / canal / irrigation flow)<br>97. Others, please specify<br>99. Refused to answer
qg2_<br>2	2	Where do you collect your drinking water?<br>1. Inside the house<br>2. Outside the house (but still in house areas)<br>3. Outside of house area<br>97. Others, please specify<br>99. Refused to answer
qg2_3	3	Do you process the water before drinking?<br>1. Yes<br>0. No<br>97. Others, please specify<br>98. Do not know<br>99. Refused to answer
qg2_4	4	If yes, how do you process the water (last time before drinking)?<br>1. Boiling<br>2. Filtered by using a water filter (eg Pureit / Nazava / Pelita etc)<br>3. Using chlorine /*Air Rahmat*/Aqua tabs<br>4. Sunbathed<br>97. Others, please specify<br>99. Refused to answer
qg2_5	5	How do you store/keep the water?<br>1. In closed container<br>2. In open container<br>3. Never store/keep water<br>97. Other please specify<br>99. Refused to answer

H. Communication Device and Access to Information

H.1. Communication device used
(Questions)

Respondent Code:……………………

Code	Mobile Phone
qh1_1	Are you currently using mobile phone?<br>[0] No<br>[1] Yes<br>[99] Refused to answer
qh1_2	Mobile phone ownership status<br>[1] personal ownership<br>[2] belongs to family member who lives in the same house<br>[3] belongs to family member who lives NOT in the same house<br>[4] belongs to others<br>[99] Refused to answer
qh1_3	How often do you use mobile phone?<br>[1] very often (almost every day)<br>[2] often (several times in a week)<br>[3] rarely (several times in a month)<br>[4] Very rarely (not necessarily once a month)<br>[99] Refused to answer
	For what purposes do you use mobile phone?<br>(select all that apply)
qh1_41	Text messages (send or receive messages)
qh1_42	Calls (send or receive calls)
qh1_43	Entertainment (songs, movies, games)
qh1_44	Shopping
qh1_45	Money transaction
qh1_46	Searching for information and news
qh1_47	Access to social media : What'sApp/ WA
qh1_48	Access to social media : Facebook
qh1_49	Access to social media : Twitter
qh1_410	Access to social media : YouTube
qh1_411	Access to social media : Instagram/ IG
qh1_412	Access to social media : Line
qcopy33oflainnya6	Others, please specify:
qh1_4_1	Do you get health information from mobile phone?<br>[0] No<br>[1] Yes<br>[99] Refused to answer
qh1_5	How do you access your mobile phone?<br>[1] independent<br>[2] need assistance from others<br>[99] Refused to answer

Code	Smart phone
qh1_6	Are you currently using Smart phone?<br>[0] No<br>[1] Yes

	[99] Refused to answer
qh1_7	Smart phone ownership status<br>[1] personal ownership<br>[2] belongs to family member who lives in the same house<br>[3] belongs to family member who lives NOT in the same house<br>[4] belongs to others<br>[99] Refused to answer
qh1_8	How often do you use Smart phone?<br>[1] very often (almost every day)<br>[2] often (several times in a week)<br>[3] rarely (several times in a month)<br>[4] Very rarely (not necessarily once a month)<br>[99] Refused to answer
	For what purposes do you use Smart phone?<br>(select all that apply)
qh1_91	Text messages (send or receive messages)
qh1_92	Calls (send or receive calls)
qh1_93	Entertainment (songs, movies, games)
qh1_94	Shopping
qh1_95	Money transaction
qh1_96	Searching for information and news
qh1_97	Access to social media : What'sApp/ WA
qh1_98	Access to social media : Facebook
qh1_99	Access to social media : Twitter
qh1_910	Access to social media : YouTube
qh1_911	Access to social media : Instagram/ IG
qh1_912	Access to social media : Line
qcopy34oflainnya6	Others, please specify:
qh1_9_1	Do you get health information from mobile phone?<br>[0] No<br>[1] Yes<br>[99] Refused to answer
qh1_10	How do you access your mobile phone?<br>[1] independent<br>[2] need assistance from others<br>[99] Refused to answer

Code	***Tablet***
qh1_11	Are you currently using Tablet?<br>[0] No<br>[1] Yes<br>[99] Refused to answer
qh1_12	Tablet ownership status<br>[1] personal ownership

	[2] belongs to family member who lives in the same house<br>[3] belongs to family member who lives NOT in the same house<br>[4] belongs to others<br>[99] Refused to answer
qh1_13	How often do you use Tablet?<br>[1] very often (almost every day)<br>[2] often (several times in a week)<br>[3] rarely (several times in a month)<br>[4] Very rarely (not necessarily once a month)<br>[99] Refused to answer
	For what purposes do you use Tablet?<br>(select all that apply**)**
qh1_141	Text messages (send or receive messages)
qh1_142	Calls (send or receive calls)
qh1_143	Entertainment (songs, movies, games)
qh1_144	Shopping
qh1_145	Money transaction
qh1_146	Searching for information and news
qh1_147	Access to social media : What'sApp/ WA
qh1_148	Access to social media : Facebook
qh1_149	Access to social media : Twitter
qh1_1410	Access to social media : YouTube
qh1_1411	Access to social media : Instagram/ IG
qh1_1412	Access to social media : Line
qcopy35oflainnya6	Others, please specify:
qh1_14_1	Do you get health information from Tablet?<br>[0] No<br>[1] Yes<br>[99] Refused to answer
qh1_15	How do you access your mobile phone?<br>[1] independent<br>[2] need assistance from others<br>[99] Refused to answer

Code	***Laptop/ Computer***
qh1_16	Are you currently using Laptop/ Computer?<br>[0] No<br>[1] Yes<br>[99] Refused to answer
qh1_17	Laptop/ Computer ownership status<br>[1] personal ownership<br>[2] belongs to family member who lives in the same house<br>[3] belongs to family member who lives NOT in the same house<br>[4] belongs to others

	[99] Refused to answer
qh1_18	How often do you use Laptop/ Computer?<br>[1] very often (almost every day)<br>[2] often (several times in a week)<br>[3] rarely (several times in a month)<br>[4] Very rarely (not necessarily once a month)<br>[99] Refused to answer
	For what purposes do you use Laptop/ Computer?<br>(select all that apply**)**
qh1_191	Text messages (send or receive messages)
qh1_192	Calls (send or receive calls)
qh1_193	Entertainment (songs, movies, games)
qh1_194	Shopping
qh1_195	Money transaction
qh1_196	Searching for information and news
qh1_197	Access to social media : What'sApp/ WA
qh1_198	Access to social media : Facebook
qh1_199	Access to social media : Twitter
qh1_1910	Access to social media : YouTube
qh1_1911	Access to social media : Instagram/ IG
qh1_1912	Access to social media : Line
qcopy36oflainnya6	Others, please specify:
qh1_19_1	Do you get health information from Laptop/ Computer?<br>[0] No<br>[1] Yes<br>[99] Refused to answer
qh1_20	How do you access your Laptop/ Computer?<br>[1] independent<br>[2] need assistance from others<br>[99] Refused to answer

Code	***Telephone***
qh1_21	Are you currently using Telephone?<br>[0] No<br>[1] Yes<br>[99] Refused to answer
qh1_22	Telephone ownership status<br>[1] personal ownership<br>[2] belongs to family member who lives in the same house<br>[3] belongs to family member who lives NOT in the same house<br>[4] belongs to others<br>[99] Refused to answer
qh1_23	How often do you use Telephone?<br>[1] very often (almost every day)

	[2] often (several times in a week)<br>[3] rarely (several times in a month)<br>[4] Very rarely (not necessarily once a month)<br>[99] Refused to answer
qh1_24	For what purposes do you use Telephone?<br>(select all that apply)
qh1_241	Text messages (send or receive messages)
qh1_242	Calls (send or receive calls)
qh1_243	Entertainment (songs, movies, games)
qh1_244	Shopping
qh1_245	Money transaction
qh1_246	Searching for information and news
qh1_247	Access to social media : What'sApp/ WA
qh1_248	Access to social media : Facebook
qh1_249	Access to social media : Twitter
qh1_2410	Access to social media : YouTube
qh1_2411	Access to social media : Instagram/ IG
qh1_2412	Access to social media : Line
qcopy37oflainnya6	Others, please specify:
qh1_24_1	Do you get health information from Telephone?<br>[0] No<br>[1] Yes<br>[99] Refused to answer
qh1_25	How do you access your Telephone?<br>[1] independent<br>[2] need assistance from others<br>[99] Refused to answer

Code	***Other communication device***
qh1_26	Are you currently using other communication device?<br>[0] No<br>[1] Yes<br>[99] Refused to answer
qh1_27	Mention other communication device
qh1_28	other communication device ownership status<br>[1] personal ownership<br>[2] belongs to family member who lives in the same house<br>[3] belongs to family member who lives NOT in the same house<br>[4] belongs to others<br>[99] Refused to answer.
qh1_29	How often do you use other communication device?<br>[1] very often (almost every day)<br>[2] often (several times in a week)<br>[3] rarely (several times in a month)<br>[4] Very rarely (not necessarily once a month)

	[99] Refused to answer
qh1_30	For what purposes do you use other communication device?<br>(select all that apply)
qh1_301	Text messages (send or receive messages)
qh1_302	Calls (send or receive calls)
qh1_303	Entertainment (songs, movies, games)
qh1_304	Shopping
qh1_305	Money transaction
qh1_306	Searching for information and news
qh1_307	Access to social media : What'sApp/ WA
qh1_308	Access to social media : Facebook
qh1_309	Access to social media : Twitter
qh1_3010	Access to social media : YouTube
qh1_3011	Access to social media : Instagram/ IG
qh1_3012	Access to social media : Line
qh1_3099	Refused to answer
qh1_3097	Other
qcopy38oflainnya6	Others, please specify:
qh1_30_1	Do you get health information from other communication device?<br>[0] No<br>[1] Yes<br>[99] Refused to answer
qh1_31	How do you access your other communication device?<br>[1] independent<br>[2] need assistance from others<br>[99] Refused to answer

How do you usually obtain information about health and nutrition? (select all that apply)
(Questions)

**Code**	**1st 1000 days of life is a golden period to support a health, smart, and high-achieving child**
qh2	Have you ever received information: 1st 1000 days of life is a golden period to support a health, smart, and high-achieving child?<br>[0] No<br>[1] Yes<br>[99] Refused to answer
qh2_1	Where did you get information about 1st 1000 days of life?<br>(select all that apply)
qh2_11	TV
qh2_12	Youtube
qh2_13	Facebook
qh2_14	Website
qh2_16	Communication, Information, and Education materials (Poster, Banner, Booklet, leaflet etc)

qh2_17	Health workers/midwife
qh2_18	Posyandu cadre
qh2_19	Class of Mother of child age below 2 (*Kelas Ibu Baduta*)
qh2_110	Class of Pregnant Mother (*Kelas Ibu Hamil)*
qh2_111	Recitation (*Pengajian*)
qh2_112	Short message or SMS
qh2_113	Magazine
qh2_199	Refused to answer
qcopy18oflainnya6	Others, please specify

**Code**	**Stunting**
qh3	Have you ever received information: Stunting?<br>[0] No<br>[1] Yes<br>[99] Refused to answer
	Where did you get information about Stunting?<br>(select all that apply)
qh3_11	TV
qh3_12	Youtube
qh3_13	Facebook
qh3_14	Website
qh3_16	Communication, Information, and Education materials (Poster, Banner, Booklet, leaflet etc)
qh3_17	Health workers/midwife
qh3_18	Posyandu cadre
qh3_19	Class of Mother of child age below 2 (*Kelas Ibu Baduta*)
qh3_110	Class of Pregnant Mother (*Kelas Ibu Hamil)*
qh3_111	Recitation (*Pengajian*)
qh3_112	Short message or SMS
qh3_113	Magazine
qh3_198	Do not know/ not remembering
qcopy39oflainnya6	Others, please specify

**Code**	**Iron Rich Food as sources of iron**
qh4	Have you ever received information: Iron Rich Food (ATIKA) as sources of iron?<br>[0] No<br>[1] Yes<br>[99] Refused to answer
	Where did you get information about Iron Rich Food (ATIKA) as sources of iron?<br>(select all that apply)
qh4_11	TV
qh4_12	Youtube
qh4_13	Facebook
qh4_14	Website
qh4_16	Communication, Information, and Education materials (Poster, Banner, Booklet, leaflet etc)
qh4_17	Health workers/midwife

qh4_18	Posyandu cadre
qh4_19	Class of Mother of child age below 2 (*Kelas Ibu Baduta*)
qh4_110	Class of Pregnant Mother (*Kelas Ibu Hamil)*
qh4_111	Recitation (*Pengajian*)
qh4_112	Short message or SMS
qh4_113	Magazine
qh4_198	Do not know/ not remembering
Qcopy40oflainnya6	Others, please specify

**Code**	**Active feeding**
qh5	Have you ever received information: Active feeding?<br>[0] No<br>[1] Yes<br>[99] Refused to answer
	Where did you get information about Active feeding?<br>(select all that apply)
qh5_11	TV
qh5_12	Youtube
qh5_13	Facebook
qh5_14	Website
qh5_16	Communication, Information, and Education materials (Poster, Banner, Booklet, leaflet etc)
qh5_17	Health workers/midwife
qh5_18	Posyandu cadre
qh5_19	Class of Mother of child age below 2 (*Kelas Ibu Baduta*)
qh5_110	Class of Pregnant Mother (*Kelas Ibu Hamil)*
qh5_111	Recitation (*Pengajian*)
qh5_112	Short message or SMS
qh5_113	Magazine
Qcopy41oflainnya6	Others, please specify

**Code**	**IFA Tablets**
qh6	Have you ever received information: IFA Tablets?<br>[0] No<br>[1] Yes<br>[99] Refused to answer
	Where did you get information about IFA Tablets?<br>(select all that apply)
qh6_11	TV
qh6_12	Youtube
qh6_13	Facebook
qh6_14	Website
qh6_16	Communication, Information, and Education materials (Poster, Banner, Booklet, leaflet etc)
qh6_17	Health workers/midwife
qh6_18	Posyandu cadre
qh6_19	Class of Mother of child age below 2 (*Kelas Ibu Baduta*)

qh6_110	Class of Pregnant Mother (*Kelas Ibu Hamil)*
qh6_111	Recitation (*Pengajian*)
qh6_112	Short message or SMS
qh6_113	Magazine
Qcopy42oflainnya6	Others, please specify

**Code**	**Nutrition for pregnant women**
qh7	Have you ever received information: Nutrition for pregnant women?<br>[0] No<br>[1] Yes<br>[99] Refused to answer
	Where did you get information about Nutrition for pregnant women?<br>(select all that apply)
qh7_11	TV
qh7_12	Youtube
qh7_13	Facebook
qh7_14	Website
qh7_16	Communication, Information, and Education materials (Poster, Banner, Booklet, leaflet etc)
qh7_17	Health workers/midwife
qh7_18	Posyandu cadre
qh7_19	Class of Mother of child age below 2 (*Kelas Ibu Baduta*)
qh7_110	Class of Pregnant Mother (*Kelas Ibu Hamil)*
qh7_111	Recitation (*Pengajian*)
qh7_112	Short message or SMS
qh7_113	Magazine
Qcopy43oflainnya6	Others, please specify

**Code**	**Handwashing using soap**
qh8	Have you ever received information: Handwashing using soap?<br>[0] No<br>[1] Yes<br>[99] Refused to answer
	Where did you get information about Handwashing using soap?<br>(select all that apply)
qh8_11	TV
qh8_12	Youtube
qh8_13	Facebook
qh8_14	Website
qh8_16	Communication, Information, and Education materials (Poster, Banner, Booklet, leaflet etc)
qh8_17	Health workers/midwife
qh8_18	Posyandu cadre
qh8_19	Class of Mother of child age below 2 (*Kelas Ibu Baduta*)
qh8_110	Class of Pregnant Mother (*Kelas Ibu Hamil)*
qh8_111	Recitation (*Pengajian*)
qh8_112	Short message or SMS

qh8_113	Magazine
Qh8_198	Do not know/ not remember
Qcopy44oflainnya6	Others, please specify

Code	Usage of Latrine
qh9	Have you ever received information: Usage of Latrine?<br>[0] No<br>[1] Yes<br>[99] Refused to answer
	Where did you get information about Usage of Latrine?<br>(select all that apply)
qh9_11	TV
qh9_12	Youtube
qh9_13	Facebook
qh9_14	Website
qh9_16	Communication, Information, and Education materials (Poster, Banner, Booklet, leaflet etc)
qh9_17	Health workers/midwife
qh9_18	Posyandu cadre
qh9_19	Class of Mother of child age below 2 (*Kelas Ibu Baduta*)
qh9_110	Class of Pregnant Mother (*Kelas Ibu Hamil)*
qh9_111	Recitation (*Pengajian*)
qh9_112	Short message or SMS
qh9_113	Magazine
Qh9_198	Do not know/ not remember
Qcopy45oflainnya6	Others, please specify

(Choices)

a. Source of information:

H.10. From these topics below, which one is easy to remember? (Please Read As Necessary and Select all answers that apply)

	H.10. From these topics below, which one is easy to remember? (Please Read As Necessary and Select all answers that apply)
**Type of Information**	
qh101	1st 1000 days of life is a golden period to support a health, smart, and high-achieving child
qh102	Stunting
qh103	Iron Rich Food as sources of iron
qh104	Active feeding
qh105	IFA Tablets
qh106	Nutrition for Pregnant Mother
qh107	Handwash using soap
qh108	Use of Latrine
qh1098	Do not know/ not remembering

qlainnya68	Others

qh11B	Have you ever discussed health and nutrition issue with your partner?
qh12B	Does your husband assist you during pregnancy?
	If you need information related to nutrition for child and mother, how do you usually obtain the information from?<br>(Select all that apply)
qh13_1TKa	Midwife
qh13_1TKb	Health workers other than midwife
qh13_1TKc	Nutritionist
qh13_1TKd	Doctor
qh13_1TKe	Nurse
qh13_1TK	Nurse / midwife / doctor / nutritionist
qh13_2K	cadre
qh13_3Kel	Friends / husband / family
qh13_4PU	Puskesmas / posyandu / puskesdes
qh13_5TV	The TV
qh13_6BK	Book
qh13_7I	Internet
qh13_97	Other (shaman, packaging label)
qh14	If you need general information about your family, what is your source of information?<br>(Select all that apply)
qh14_1	Cadre/ Nurse / midwife / doctor / nutritionist
qh14_2	cadre
qh14_3	Friends / husband / family
qh14_4	Puskesmas / posyandu / puskesdes
qh14_5	The TV
qh14_6	Book
qh14_7	Internet
qh14_97	Other (shaman, toga toma)
